# High Prevalence of Osteopenia and Osteoporosis in Total Hip and Total Knee Arthroplasty Patients and Effects of Anti-Resorptive Agents on Bone Health Optimization: A Systematic Review and Meta-Analysis

**DOI:** 10.3390/jcm14248769

**Published:** 2025-12-11

**Authors:** Ronald Man Yeung Wong, Pui Yan Wong, Joon Kiong Lee, Aasis Unnanuntana, Tanawat Amphansap, Peter R. Ebeling, Jacqueline Close, Gustavo Duque, Sheung Wai Law, Wing Hoi Cheung

**Affiliations:** 1 Department of Orthopaedics & Traumatology, The Chinese University of Hong Kong, Hong Kong, China; 2 Department of Orthopedic Surgery, Beacon Hospital, Petaling Jaya 46050, Selangor, Malaysia; 3 Department of Orthopaedic Surgery, Faculty of Medicine Siriraj Hospital, Mahidol University, Bangkok 10700, Thailand; 4 Department of Orthopedics, Police General Hospital, Bangkok 10400, Thailand; 5 Department of Medicine, School of Clinical Sciences, Monash University, Clayton, VIC 3168, Australia; 6 School of Clinical Medicine, University of New South Wales, Sydney, NSW 2052, Australia; 7 Bone, Muscle & GeroScience Research Group, Research Institute of the McGill University Health Centre, Montreal, QC H3H 2R9, Canada

**Keywords:** arthroplasty, bone health optimization, osteoporosis, periprosthetic fracture, revision surgery

## Abstract

**Background**: Osteoarthritis is a leading cause of chronic pain and long-term disability in adults, which commonly affects the hip and knee joints. Joint arthroplasties are one of the management strategies for end-stage osteoarthritis. Periprosthetic fractures after hip or knee arthroplasties have mortality rates comparable to hip fractures. Recent studies assessed bone health optimization and the use of anti-osteoporotic agents in elective hip and knee arthroplasty surgeries. This systematic review and meta-analysis aimed to determine the prevalence of osteoporosis before surgery and the effect of bone health optimization on periprosthetic fractures and revisions. **Methods**: A systematic search was carried out on three databases, including PubMed, Embase, and Web of Science. The keywords used were (Revision or Periprosthetic fracture) AND (osteop*) and (Total Knee* or Total Hip*). Studies that included subjects aged >50 years with investigated outcomes were included in the review. The quality of selected randomized controlled trials was assessed using the Cochrane Collaboration tool, and non-randomized studies were assessed using the Newcastle–Ottawa Scale. The review was not registered with the International Prospective Register of Systematic Reviews (PROSPERO). **Results**: A total of 2482 records were identified. Twenty-three studies were included, and eighteen were used for quantitative analysis. Pooled overall prevalence of osteopenia in patients undergoing total knee arthroplasty (TKA)/total hip arthroplasty (THA) surgery was 42.87% (95% confidence interval (CI) 32.65 to 53.09). Pooled overall prevalence of osteoporosis in patients undergoing TKA/THA surgery was 23.99% (95% CI 15.72 to 32.26). The overall mean difference was in favor of anti-resorptive treatment on periprosthetic BMD of the medial calcar region (Gruen zone 7) after THA (12.16% (95% CI 8.78 to 15.53, *p* < 0.00001). Pooled odds ratio of periprosthetic fracture was 1.27 (95% CI 1.08 to 1.48, *p* = 0.003) in favor of the control group compared to bisphosphonate treatment. The pooled hazard ratio for all-cause revisions after TKA/THA for both osteopenia and osteoporotic patients was 0.26 (95% CI 0.13 to 0.51, *p* = 0.0001, I^2^ 76%), signifying an improvement with bisphosphonates. Limitations of this study include the heterogeneity and retrospective nature of the included studies, with the average level of evidence subject to bias. **Conclusions**: There was a high prevalence of osteopenia/osteoporosis amongst patients undergoing total knee and total hip arthroplasty at 66.86%. Whilst bone health optimization with bisphosphonates may decrease the risk of revisions, the risk of periprosthetic fracture appeared to increase. Further research will be required to evaluate the effects of bone health optimization on the risk of periprosthetic fracture and revisions, and the effects of anabolic agents on periprosthetic fractures.

## 1. Introduction

Osteoarthritis is a leading cause of chronic pain and long-term disability in adults, which commonly affects the hip and knee joints [1]. Studies have shown that osteoarthritis is a complex disease characterized by pathological changes in the joint. There is increasing evidence that low-grade synovitis contributes to the radiographic progression of pain in osteoarthritis [2,3]. Given the aging population, the demand on healthcare systems for patients is expected to rise globally, and this includes joint arthroplasties, which are one of the most successful management strategies for end-stage osteoarthritis. These surgical procedures are now commonly performed worldwide, and have provided pain relief and improvement in quality of life and function for many patients [4,5]. In fact, there are more than 1 million total hip replacements performed worldwide each year [4], and in the United States (US) alone, 700,000 total knee arthroplasties are performed per year [5]. Due to the increasing incidence of these procedures, complications have also been on the rise, including periprosthetic fractures and revisions, imposing a substantial burden on the healthcare system.

Periprosthetic fractures associated with arthroplasties have increased exponentially over the years, with rates reaching 15% in major joint registries [6]. More importantly, similar to hip fractures [7], most of these cases result from low-energy trauma, accounting for 75% of fractures. Osteoporosis affects more than 500 million people worldwide, and studies have now stated that periprosthetic fractures may be the next fragility fracture epidemic [8]. Periprosthetic fractures may often require surgical fixation or revision of the implant, which can be complex with prolonged operation times, and periods of restricted weight bearing can negatively impact the level of function. Unfortunately, periprosthetic fractures after hip or knee arthroplasties have mortality rates comparable to hip fractures as well [9,10]. Recent articles have also reported higher revision rates for total knee and hip arthroplasties in patients with osteoporosis [11,12]. Performing revision operations in osteoporotic bone poses a major challenge amongst orthopedic surgeons. Therefore, the prevention of both periprosthetic fractures and revision in both total knee arthroplasty (TKA) and total hip arthroplasty (THA) is important.

Many initiatives have been placed in the establishment of Fracture Liaison Services to treat osteoporosis in fragility fracture patients to prevent secondary fractures and decrease mortality [13,14]. However, in the context of total hip and knee arthroplasties, the rationale of bone health optimization has only recently been introduced [15]. Numerous studies have recently been published assessing bone health and the use of anti-osteoporotic agents before elective hip and knee arthroplasty surgeries. The objective of this study was to perform a systematic review and meta-analysis based on the existing literature to determine (i) the prevalence of osteoporosis in patients aged >50 years indicated for total knee or hip arthroplasty and (ii) the effect of bone health optimization compared to a lack of bone health optimization on periprosthetic fractures and revisions in patients aged >50 years with total knee and hip arthroplasties.

## 2. Materials and Methods

### 2.1. Search Strategy

A systematic search was conducted on three databases, including PubMed, Embase, and Web of Science, with the last access date on 27 March 2025. The keywords for the search were (Revision or Periprosthetic fracture) AND (osteop*) and (Total Knee* or Total Hip*). The detailed search strategy is shown in Supplementary Table S1. The literature search was limited to studies in the English language, and no restriction was applied on publication dates. In addition, reference lists on relevant articles were searched manually for potentially eligible articles. The review was not registered with the International Prospective Register of Systematic Reviews (PROSPERO) due to time limitations. This systematic review and meta-analysis were reported based on the guidelines of the Preferred Reporting Items for Systematic Reviews and Meta-analyses (PRISMA) statements (Supplementary File S1) [16] despite no PROSPERO registration.

### 2.2. Search Criteria

The inclusion criteria were (1) subjects aged ≥50 years with elective primary TKA/THA who either studied (2) the prevalence of osteopenia/osteoporosis before surgery or (3) the effects of anti-resorptive drugs on bone health after surgery. The exclusion criteria were (1) no full text being available; (2) articles not being in English; (3) research taking the form of a conference abstract; (4) the inclusion of a non-elective primary TKA/THA surgery to treat other indications, e.g., femoral neck fracture; (5) not using the World Health Organization (WHO) classification for osteoporosis diagnosis (DXA scan); (6) studies with a low level of evidence including case–control studies; and (7) non-randomized studies which investigated the effects of anti-resorptive in subjects with normal BMD as the effects of anti-resorptive agents for the treatment of subjects with low BMD was the main objective of this study.

### 2.3. Selection of Studies

Article selection was conducted by two independent reviewers (PYW, CHT). All references identified with the defined keywords from the three databases were imported into a reference management tool, and duplicate references were removed after combining the databases. References were screened for eligibility by title and abstract based on the established inclusion and exclusion criteria. The full-text articles of the potentially eligible references were then retrieved for further assessment. Disagreements during article selection were resolved by consensus. If discrepancies still occurred, a third reviewer (RMYW) made the final decision.

### 2.4. Data Extraction

Data extraction of the included articles was conducted by two independent reviewers (PYW, MKF) and was presented in a standardized table form. The following data were extracted: (1) study characteristics including the last name of the first author, year of publication, country/region of the study, study design and end point of the study; (2) subject characteristics including sample size (N), age (mean ± standard deviation), gender, surgical indication, type of surgery, history of osteoporosis drug use and its period of use, dual-energy X-ray absorptiometry (DXA) scan timing and skeletal site scanned; and (3) outcomes including prevalence of osteopenia/osteoporosis, incidence and time of periprosthetic fracture after surgery, cause of arthroplasty revision, mortality and other surgical outcomes and complications.

### 2.5. Quality Assessment of Studies

The quality of the RCT studies was assessed by the two independent reviewers (PYW, MKF) using the Cochrane Collaboration tool, in which the risk of bias was assessed in the following domains: random sequence generation, allocation concealment, blinding of participants and personnel, blinding of outcome assessments, incomplete outcome data, and selective reporting and other sources of bias. The risk of bias of each domain was classified as “low risk”, “high risk”, or “unclear” [17]. For non-randomized studies, the Newcastle–Ottawa Scale (NOS) was used to assess the quality [18] of the included cohort studies, in which a maximum of 4 points was assigned for selection, 2 points were assigned for comparability, and 3 points were assigned for the assessment of outcomes. In addition, the NOS adapted for cross-sectional studies assigns a maximum of 5 points for selection, 1 point for comparability, and 3 points for the assessment of outcomes [19]. Overall quality was divided into low quality (0–3), medium quality (4–6), and high quality (7–9), respectively. Disagreements during the quality assessment of studies were resolved by consensus between the two independent reviewers. If discrepancies still occurred, a third reviewer (RMYW) made the final decision.

### 2.6. Data Analysis

Assessment outcomes, including prevalence of osteopenia/osteoporosis and the effects of the anti-resorptive drug on the surgical outcomes, were analyzed. Quantitative analyses were performed if at least 2 studies were available. The prevalence of osteopenia/osteoporosis before surgery was calculated as the number of subjects with DXA-diagnosed osteopenia or osteoporosis/total number of people with DXA scans. The effects of anti-resorptive drugs included the changes in periprosthetic bone mineral density (BMD), incidence of periprosthetic fracture, revision surgery, and mortality. To compare between the anti-resorptive treatment group and the control group, only non-randomized studies considering subjects with osteopenia/osteoporosis only and RCT studies were included for meta-analysis. The odds ratio, hazard ratio, mean difference, and their 95% CI were computed using the generic inverse variance method (the calculated confidence interval may be slightly different from that provided in the original papers). Review Manager (RevMan 5.4, The Cochrane Centre, The Cochrane Collaboration, Copenhagen, Denmark) was used for all analyses and production of forest plots. A *p*-value < 0.05 was considered statistically significant for the Z-test. Heterogeneity was evaluated by the Q-test with the I^2^ index, where an I^2^ index > 50% or *p*-value < 0.1 represented significant heterogeneity between the included studies. If there was no significant heterogeneity, a fixed-effects model was used. If there was significant heterogeneity, a random-effects model was used. Subgroup analysis was performed in the pooled analyses of prevalence of osteopenia/osteoporosis before TKA/THA surgery to investigate the effects of these factors on the heterogeneity among studies. Sensitivity analysis was performed by removing studies of low quality and leaving these studies out to assess the robustness of the analyses. Potential publication bias was evaluated for meta-analysis, including 10 studies or more, through the visual inspection of the symmetry of the funnel plots, and Egger’s test was also performed; *p* < 0.1 was considered statistically significant. To adjust for the publication bias, the trim-and-fill method was used. The R package (R core team, 2025, version R 4.5.2, Vienna, Austria) was used for publication bias assessments. Qualitative analysis was performed if the data was not enough (fewer than 2 studies) to be included in the meta-analysis or with missing data, which were excluded from the quantitative analysis.

## 3. Results

### 3.1. Search Results

**Figure 1:**
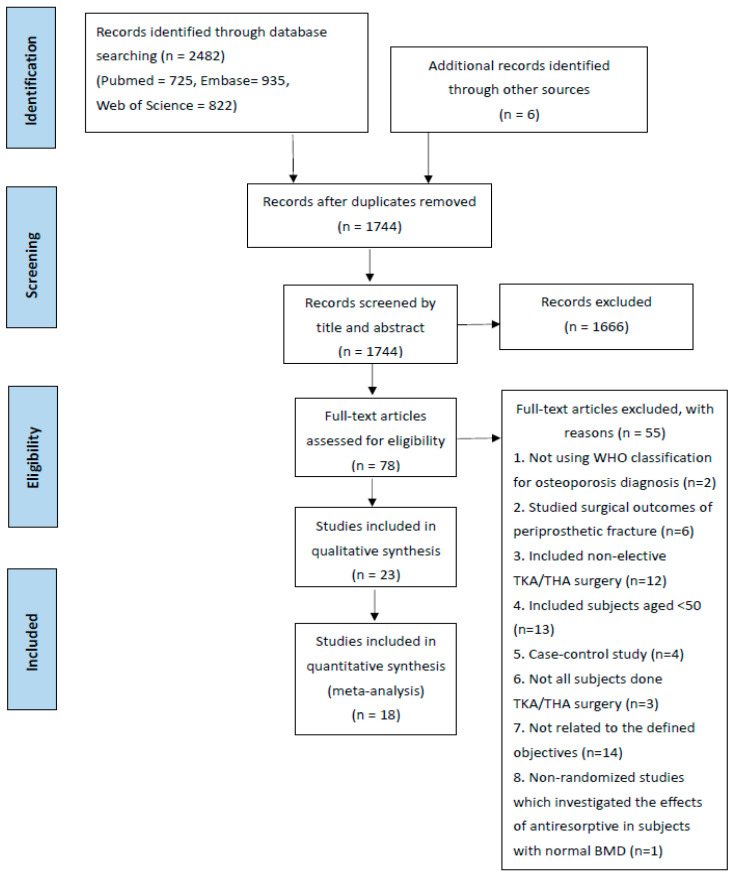
Prisma chart.

A total of 2482 records were identified through database searching (n = 2482), and 6 additional records were identified through manual searching. Duplicate records were removed, and 1744 records remained. The 1744 records were then screened by title and abstract, and 1666 records were excluded based on the inclusion and exclusion criteria. The remaining 78 articles were retrieved in their full text and were assessed for eligibility. A total of 55 articles were excluded, including articles which studied surgical outcomes for periprosthetic fracture without investigating the effects of an anti-resorptive agent (n = 6); not all patients received TKA/THA surgery so the overall effects of anti-resorptive agents could not be clearly determined (n = 3), and finally 23 articles were included for qualitative analysis, of which 18 studies were included for meta-analysis (Figure 1).

### 3.2. Study Characteristics

In total, 23 articles [20,21,22,23,24,25,26,27,28,29,30,31,32,33,34,35,36,37,38,39,40,41,42] were published from 2003 to 2024. This included 8 studies [24,25,26,27,28,29,30,31] with randomized controlled trials, 13 cohort studies (2 prospective [22,23] and 11 retrospective studies [32,33,34,35,36,37,38,39,40,41,42]), and 2 cross-sectional studies [20,21]. Ten articles studied the prevalence of osteopenia/osteoporosis before primary TKA/THA surgery [20,21,23,33,37,38,39,40,42,43] and thirteen articles studied the effects of anti-resorptive drugs on bone health after primary TKA/THA surgery [22,24,25,26,27,28,29,30,31,32,34,35,36]. In total, 11 articles included subjects from Asian countries/regions [22,23,24,25,26,27,28,31,33,37,41] and 12 articles included subjects from Western countries/regions [20,21,29,30,32,34,35,36,38,39,40,42]. There were 11 articles that evaluated subjects who underwent TKA surgery only [21,22,23,24,25,34,36,37,38,39,41], 10 articles that evaluated subjects who underwent THA surgery only [26,27,28,29,30,31,32,33,35,40], and 2 articles that evaluated subjects who underwent either TKA or THA surgery [20,42]. Finally, 7 articles included women only [21,23,24,25,29,30,41] and the remaining 16 articles included both genders [20,22,26,27,28,31,32,33,34,35,36,37,38,39,40,42]. Detailed characteristics of the included studies are summarized in Supplementary Tables S2 and S3.

### 3.3. Quality of Selected Studies

**Figure 2:**
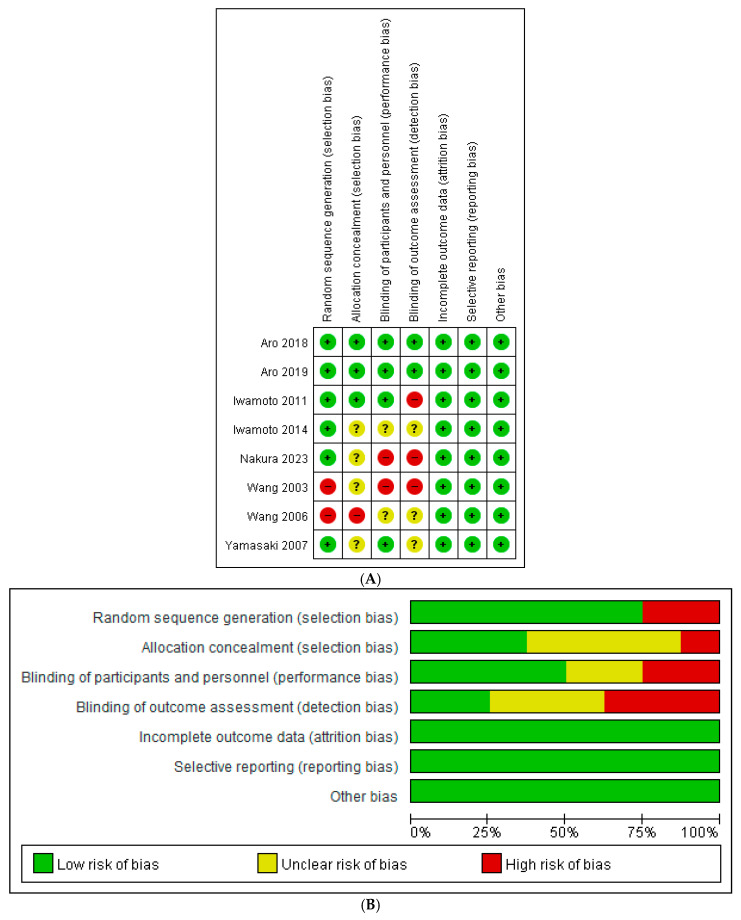
(**A**) Risk of bias graph for included randomized controlled trials [24,25,26,27,28,29,30,31]. (**B**) Risk of bias summary for included randomized controlled trials.

The Cochrane risk of bias tool was used to assess risk of bias for the 8 RCT studies. The risk of bias graph and risk of bias summary are shown in Figure 2. The NOS was used to assess the risk of bias for the 15 non-randomized studies and was summarized in Table 1 (cohort studies) with a score (Mean ± SD) of 7.46 ± 1.39 and Table 2 (cross-sectional studies) with a score of 7.5 ± 0.71, respectively. All studies were of medium-to-high quality and were suitable for quantitative and qualitative analysis.

### 3.4. Prevalence of Osteopenia/Osteoporosis in Patients Undergoing a TKA/THA

**Figure 3:**
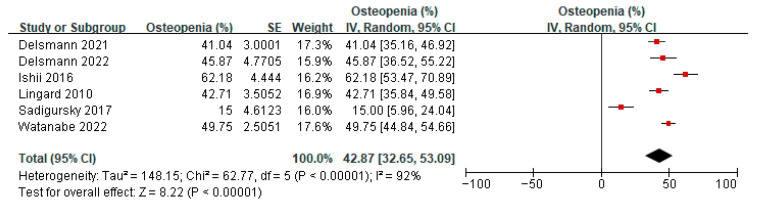
Prevalence of osteopenia before TKA/THA surgery [20,21,23,33,38,39].

**Figure 4:**
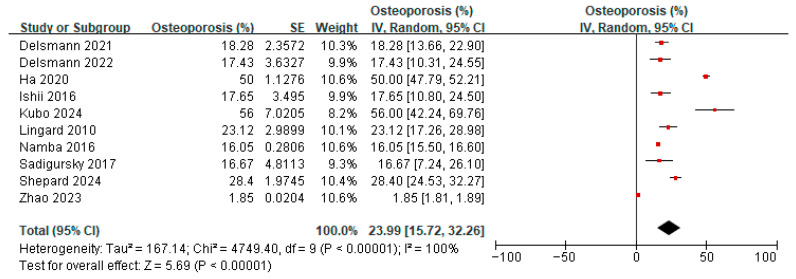
Prevalence of osteoporosis before TKA/THA surgery [20,21,23,36,37,38,39,40,41,42].

Six studies [20,21,23,33,38,39] reported on the prevalence of osteopenia amongst patients before TKA or THA. A meta-analysis of these six studies showed the overall prevalence of osteopenia to be 42.87% (95% confidence interval (CI) 32.65 to 53.09, *p* < 0.00001, I^2^ 92%) (Figure 3). Ten studies [20,21,23,33,37,38,39,40,41,42] reported on the prevalence of osteoporosis amongst patients before TKA or THA. A meta-analysis of these 10 studies showed the overall prevalence of osteoporosis to be 23.99% (95% CI 15.72 to 32.26, *p* < 0.00001, I^2^ 100%) (Figure 4). The prevalence of both osteopenia and osteoporosis was 66.86%, signifying a high percentage of patients with low bone mineral density (BMD). Subgroup analysis for the prevalence of osteopenia before TKA/THA surgery by study design showed the overall prevalence of osteopenia to be 29.02% (1.86–56.17, *p* = 0.04) in cross-sectional studies compared to 49.40% (41.40–57.40) in cohort studies (Supplementary Figure S1). When analyzed by country/region, the results showed that compared to Western locations (36.33%, 95% CI: 24.25–48.41), the prevalence of osteopenia in Asian locations tended to be higher (55.42%, 95% CI: 43.29–67.56) (Supplementary Figure S2). Subgroup analysis for the prevalence of osteoporosis before TKA/THA surgery by study design showed a similar effect on the estimate (Supplementary Figure S3). When analyzed by country/region, the prevalence of osteoporosis in the Asian locations tends to be higher (40.90%, 95%CI: 17.20–64.59) compared to Western locations (17.29%, 95% CI: 9.36–25.21) (Supplementary Figure S4).

Sensitivity analysis was conducted for the prevalence of osteopenia before TKA/THA surgery; after removing one study [21] where the results differed greatly from others, the heterogeneity decreased from 92% to 78%. The direction of the effect estimate remained unchanged, and the results remained statistically significant. Other analyses showed similar effects regarding the change in heterogeneity, direction of estimate, and significance (Supplementary Table S4). For the sensitivity analysis of the prevalence of osteoporosis, most of the analyses did not show changes in heterogeneity and the direction of estimates, and significance also remained unchanged (Supplementary Table S5).

### 3.5. Effect of Anti-Resorptive Agents on Periprosthetic Bone Mineral Density After TKA/THA

Four studies [26,27,29,30] reported on the effect of anti-resorptive agents compared to changes in periprosthetic BMD in the medial calcar region (Gruen zone 7) after THA: one study used denosumab (12 months) compared with a placebo [30], one study used zoledronic acid (12 months) compared with a placebo [29], and two studies used oral bisphosphonates (48 weeks of alendronate compared with no medication [28] and 6 months of risedronate compared with calcium lactate [26]). A meta-analysis of these four studies showed that the overall mean difference in periprosthetic BMD in the medial calcar region (Gruen zone 7) after THA was 12.16% greater in those undergoing anti-resorptive treatment (95% CI 8.78 to 15.53, *p* < 0.00001) compared to the control group (Supplementary Figure S5).

Nakura et al. found that at 24 months after THA, comparing denosumab and risedronate, denosumab led to an increase in BMD (+5.9%) whilst risedronate caused a significant decrease in BMD (−19.2%) in Zone 7 [31]. Iwamoto et al. found that alendronate and combined therapy (alendronate and alfacalcidol) prevented periprosthetic BMD loss around femoral implants after THA compared with no medication, but no significant differences were shown between the alendronate and combined therapy groups at 48 weeks [28]. Murahashi et al. showed that at 12 months after TKA, denosumab significantly improved BMD (+0.7%) in the proximal medial tibial bone of the periprosthetic region, whilst BMD in the control group decreased significantly (−19.7%) after adjusting for confounding factors, including age and the preoperative femorotibial angle [22].

### 3.6. Effect of Bisphosphonates on Periprosthetic Fracture After TKA/THA

**Figure 5:**
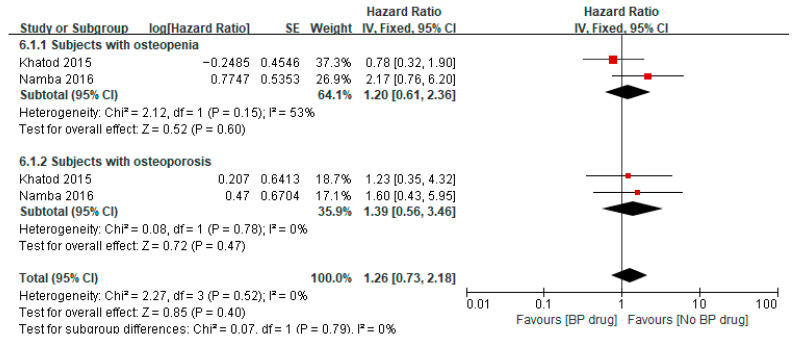
Hazard ratio of periprosthetic fracture after surgery in subjects aged ≥65 years, comparing to bisphosphonate (BP) drug users to non-bisphosphonate drug users [32,36].

Two studies [32,36] reported on the hazard ratio of bisphosphonates on periprosthetic fracture after TKA/THA in patients with osteopenia, where both studies used bisphosphonates for a minimum of 6 months. Meta-analysis of these two studies showed a pooled hazard ratio of 1.20 (95% CI 0.61 to 2.36, *p* = 0.60, I^2^ 53%), indicating that among patients with osteopenia, the hazard of periprosthetic fracture was 1.20 times higher in patients receiving bisphosphonates compared to the control group (Figure 5). The two studies [32,36] also reported on the hazard ratio of bisphosphonates on periprosthetic fracture after TKA/THA in patients with osteoporosis. A meta-analysis of these two studies showed a pooled hazard ratio of 1.39 (95% CI 0.56 to 3.46, *p* = 0.47, I^2^ 0%), indicating that among patients with osteoporosis, the hazard of periprosthetic fracture was 1.39 times higher in patients receiving bisphosphonates compared to the control group. The pooled hazard ratio on periprosthetic fracture after TKA/THA for both osteopenia and osteoporotic patients was 1.26 (95% CI 0.73 to 2.18, *p* = 0.40, I^2^ 0%), indicating that combining patients with osteopenia and osteoporosis, the hazard of periprosthetic fracture was 1.26 times higher in patients receiving bisphosphonates compared to the control group. However, the results of the pooled hazard ratios were not statistically significant as illustrated by the diamond (pooled estimate and 95% CI) crossing the line of null effect (Figure 5).

**Figure 6:**
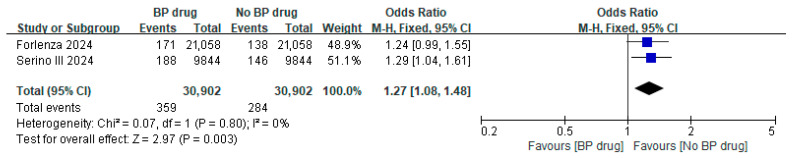
Odds ratio of periprosthetic fracture in subjects with osteoporosis, comparing bisphosphonate (BP) drug users to non-bisphosphonate drug users at 2 years postoperatively [34,35].

Two studies [34,35] reported on the odds ratio of bisphosphonates on periprosthetic fracture after TKA/THA in patients with osteoporosis 2 years post-surgery; both studies used bisphosphonates for a minimum of 6 months. The odds ratios from these two studies were controlled for age, sex, comorbidities, and other risk factors in the multivariate analysis [34,35]. A meta-analysis of these two studies showed a pooled odds ratio of 1.27 (95% CI 1.08 to 1.48, *p* = 0.003, I^2^ 0%) with the pooled estimate and 95% CI not crossing the line of null effect, indicating that the odds of periprosthetic fracture in patients receiving bisphosphonate were significantly 1.27 times higher than those in the control group (Figure 6). In addition, Forlenza et al. showed that among patients with osteoporosis and without bisphosphonate treatment, the odds of a periprosthetic fracture with cementless TKA was 10.40 (95% CI 1.98 to 191.63) (*p* = 0.026) times higher than the cemented TKA group, while the related odds ratio in bisphosphonate users was not significant (OR = 1.37, 95% CI: 0.30 to 7.17, *p* = 0.682) [34]. An RCT study conducted by Aro et al. found that one patient from the placebo group underwent internal fixation of a periprosthetic fracture 9 years after THA, but the total number of patients that survived in each group at that time-point was not specified; therefore, the odds ratio was not calculated [29]. Two studies investigated the effects of a 6-month course of oral alendronate after THA, and no periprosthetic fracture was observed in either the alendronate group or the control group at 1 year [24] and 3 years [25] after surgery, respectively.

### 3.7. Effect of Bisphosphonates on All-Cause Revisions After TKA/THA

**Figure 7:**
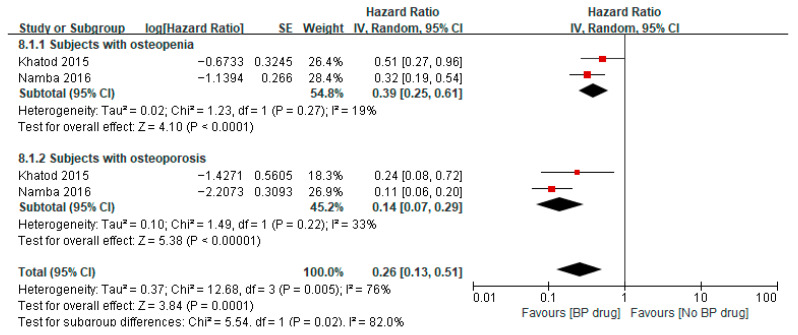
Hazard ratio of all-cause revisions after surgery in subjects aged ≥65 years, comparing bisphosphonate (BP) drug users to non-bisphosphonate drug users [32,36].

**Figure 8:**
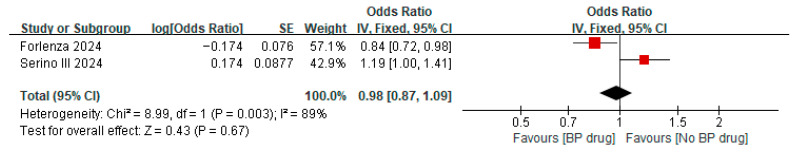
Odds ratio of all-cause revisions in subjects with osteoporosis, comparing bisphosphonate (BP) drug users to non-bisphosphonate drug users at 2 years postoperatively [34,35].

Two studies [32,36] reported on the hazard ratio of bisphosphonates for all-cause revisions after TKA/THA in patients with osteopenia, where both studies used bisphosphonates for a minimum of 6 months. A meta-analysis of these two studies showed a pooled hazard ratio of 0.39 (95% CI 0.25 to 0.61, *p* < 0.0001, I^2^ 19%), indicating that among patients with osteopenia, the hazard of all-cause revisions was significantly 0.39 times lower in the bisphosphonate group than that in the control group (Figure 7). The two studies [32,36] also reported on the hazard ratio of bisphosphonates for all-cause revisions after TKA/THA in patients with osteoporosis. A meta-analysis of these two studies showed a pooled hazard ratio of 0.14 (95% CI 0.07 to 0.29, *p* < 0.00001, I^2^ 33%), indicating that among patients with osteoporosis, the hazard of all-cause revisions was significantly 0.14 times lower in the bisphosphonate group than in the control group. The pooled hazard ratio for all-cause revisions after TKA/THA for both osteopenia and osteoporotic patients was 0.26 (95% CI 0.13 to 0.51, *p* = 0.0001, I^2^ 76%) with the pooled estimate and 95% CI not crossing the line of null effect, indicating that combining patients with osteopenia and osteoporosis, the hazard of all-cause revisions was significantly 0.26 times lower in the bisphosphonate group than in the control group (Figure 7). Two studies [34,35] reported on the odds ratio of bisphosphonates on all-cause revisions after TKA/THA in patients with osteoporosis. A meta-analysis of these two studies showed a pooled odds ratio of 0.98 (95% CI 0.87 to 1.09, *p* = 0.67, I^2^ 89%), indicating that among patients with osteoporosis, the odds of all-cause revisions for patients receiving bisphosphate were 0.98 times lower than in the control group, despite the results not being statistically significant as illustrated by the pooled estimate and 95% CI crossing the line of null effect (Figure 8).

### 3.8. Effect of Bisphosphonates on Aseptic Revisions After TKA/THA

Two studies [32,36] reported on the hazard ratio of bisphosphonates for aseptic revisions after TKA/THA in patients with osteopenia, where both studies used bisphosphonates for a minimum of 6 months. A meta-analysis of these two studies showed a pooled hazard ratio of 0.50 (95% CI 0.31 to 0.81, *p* = 0.005, I^2^ 0%), indicating that among patients with osteopenia, the hazard of aseptic revisions in the bisphosphate group was significantly 0.50 times lower than in the control group (Supplementary Figure S6). The two studies [32,36] also reported on the hazard ratio of bisphosphonates on aseptic revisions after TKA/THA in patients with osteoporosis. A meta-analysis of these two studies showed a pooled hazard ratio of 0.18 (95% CI 0.09 to 0.38, *p* < 0.00001, I^2^ 27%), indicating that among patients with osteoporosis, the hazard of aseptic revisions in the bisphosphate group was significantly 0.18 times lower than in the control group. The pooled hazard ratio on aseptic revisions after TKA/THA for both osteopenia and osteoporotic patients was 0.32 (95% CI 0.17 to 0.63, *p* = 0.0008, I^2^ 66%), indicating that when combining patients with osteopenia and osteoporosis, the hazard of aseptic revisions in the bisphosphate group was significantly 0.32 times lower than in the control group (Supplementary Figure S6).

Two studies [34,35] reported on the odds ratio of anti-resorptive agents on aseptic revisions after TKA/THA in patients with osteoporosis. A meta-analysis of these two studies showed a pooled odds ratio of 1.01 (95% CI 0.85 to 1.21, *p* = 0.90, I^2^ 47%), indicating that the odds of aseptic revisions in patients receiving anti-resorptive agents were 1.01 times higher than those in the control group. However, this result was not statistically significant (Supplementary Figure S7).

### 3.9. Effect of Bisphosphonates on Mortality After TKA/THA

Two studies [34,35] reported on the odds ratio of anti-resorptive agents on mortality after TKA/THA in patients with osteoporosis, where both studies used bisphosphonates for a minimum of 6 months. A meta-analysis of these two studies showed a pooled odds ratio of 1.74 (95% CI 0.22 to 14.09, *p* = 0.60, I^2^ 79%), indicating that the odds of mortality in patients receiving anti-resorptive agents were 1.74 times higher than in the control group (Supplementary Figure S8).

### 3.10. Publication Bias

The assessment of publication bias was conducted for the pooled analysis of prevalence of osteoporosis before TKA/THA surgery, which included 10 studies. The funnel plot for the pooled proportion did not show any sign of symmetry (Supplementary Figure S9). The test for funnel plot asymmetry using Egger’s test showed statistical significance (*p* < 0.1), indicating signs of asymmetry for the funnel plot. After using trim-and-fill methods to detect and adjust for publication bias, no estimated number of missing studies was imputed, and the results of the meta-analysis remained the same, suggesting the possibility that the asymmetry of the funnel plot was caused by reasons other than publication bias.

## 4. Discussion

The prevalence of low BMD, including osteopenia and osteoporosis, was high in our study at 66.86%; it is known that high rates are associated with periprosthetic fractures and revision operations in total knee and hip arthroplasties. Periprosthetic fractures are now increasingly frequent, and previous studies have even reported clinical outcomes in patients aged 90 and above with operative treatment, signifying the impact of the aging population [44]. Although conservative management may be indicated in some cases of periprosthetic hip and knee fractures, the large majority require surgery for pain relief and early mobilization. These operations are often challenging and require experienced surgeons due to complex fracture patterns, major blood loss, and poor bone quality encountered during surgery. To increase the complexity, previous studies have also shown that osteoporosis is associated with fracture non-union [45], which is also evidenced in pre-clinical studies showing how osteoporotic fracture healing is compromised in various phases, leading to research in novel methods for acceleration [46,47,48,49]. More importantly, most periprosthetic fractures are fragility fractures that occur in older people who have multiple comorbidities and, therefore, even if the operation is performed, the patient may experience postoperative complications such as hospital-acquired pneumonia, which can increase mortality [50]. Therefore, a multidisciplinary approach and dedicated pathways, including medical optimization and careful surgical planning, are often required [8]. For revision arthroplasty, there is also an increased risk of periprosthetic fractures, implant migration and loosening with low BMD [51].

Bone health optimization is a recent concept that includes bone status assessment, identification and correction of secondary causes, and initiation of treatment as needed due to the increasing evidence of poor bone health being associated with poor clinical outcomes [15,52]. In fact, it has been shown that for elective spinal surgery, osteoporosis-related complications, including kyphosis, hardware failure, and fractures, can occur in 50% of patients [15]. Furthermore, a previous study also showed that in patients who were 50 years or older and were candidates for arthroplasty and thoracolumbar surgery, 91% of all patients met the threshold for osteoporosis treatment, signifying the potential importance of preoperative optimization for bone health. Due to this, it has been recommended in an American Orthopaedic Association (AOA) critical issue article that patients 50 years or older undergoing major orthopedic surgery should be considered for bone health optimization [12,50]. This practical model is similar to fracture liaison services [13,14,53] where preoperative bone health optimization can potentially improve clinical outcomes and reduce complications and costs. In theory, bone health optimization for osteopenia and osteoporotic patients may be able to decrease revisions and periprosthetic fractures for arthroplasty patients and reduce complications in spinal surgeries, which are often elective operations. Recently, a bone health optimization framework was introduced by the Malaysian Bone Health Optimization Network to improve surgical outcomes and patient care [54].

Low BMD is commonly identified preoperatively amongst patients who plan for elective joint operations [55], as was found in our review, with prevalence of osteopenia reaching 42.87% (95% CI 32.65–53.09) and prevalence of osteoporosis reaching 23.99 (95% CI 15.72–32.26). However, Egger’s test for the asymmetry of the funnel plot showed statistical significance, and no estimated number of missing studies was imputed in the trim-and-fill method. The discrepancy between Egger’s test and the trim-and-fill method could be due to the possibility that the asymmetry of the funnel plot was caused by other reasons, such as heterogeneity among the studies, rather than publication bias, given the high heterogeneity among the studies. In this systematic review and meta-analysis, the pooled hazard ratios and odds ratios of different surgical outcomes were examined. The hazard ratio is interpreted as the instantaneous event rate at any time during follow-up among exposed individuals compared to control individuals and is assumed to be constant over time. On the other hand, the odds ratio is interpreted as the odds of an outcome given an exposure of interest to the odds of outcomes in the absence of that exposure of interest. The pooled hazard ratio for all-cause revisions (0.26 (0.13–0.51)) and aseptic revisions (0.32 (0.17–0.63)) in bisphosphonate users compared to non-bisphosphonate users indicated that the relative rate of all-cause revisions and aseptic revisions was significantly lower in bisphosphonate users. The odds ratio of all-cause revisions and aseptic revisions showed no significant differences between the two groups. The bisphosphonate agents underwent a significant improvement in revision rates compared to no treatment. This is expected as BMD surrounding the implant is improved, which is especially relevant in the use of cementless implants. Surprisingly, our results showed that the odds ratio with the use of bisphosphonate treatment for periprosthetic fractures favored the control group, although the hazard ratio showed no significant differences. The discrepancies of significance between the odds ratio and hazard ratio could be due to the heterogeneity of the included studies; for instance, two articles which reported odds ratio investigated a total of 2-year outcomes postoperatively only in patients undergoing elective TKA/THA surgery [34,35], whilst two articles which reported hazard odds had a mean follow-up of more than 2 years in patients undergoing elective TKA/THA surgery diagnosed with osteoarthritis only [32,36]. However, we should also be cautious regarding the interpretation, as there is an inherent limitation of retrospective studies as well as the possibility of selection bias; furthermore, both the time and period for the use of bisphosphonate treatment were not consistent. Serino III et al. showed that the increased risk of periprosthetic fractures and atypical femur fractures in preoperative bisphosphonate users may share the same underlying mechanism, in which bisphosphate prevented the repair of microdamage sustained intraoperatively and also inhibited the bone turnover necessary for osseointegration of uncemented implants, potentially indicating a suppressed healing response in bisphosphonate users after surgery [35]. A previous study postulated that a higher rate of periprosthetic fracture in the anti-resorptive group is due to intraoperative mechanical and thermal damage, which can lead to temporary periprosthetic bone resorption that requires around 3 months to heal, and bisphosphonates can potentially suppress this healing [35]. Other recent studies also showed similar findings with an increased association of periprosthetic fractures and the use of bisphosphonate treatment [56,57]. However, further studies are required to verify this, as these studies do not show causative effects, and the mechanism is not confirmed. While anabolic agents such as romosozumab or teriparatide may accelerate bone formation, their role in the perioperative setting warrants further investigation, particularly as they were not the focus of this review [58].

Furthermore, confounding factors that may affect the association of bisphosphonates and periprosthetic fractures should be considered. Previous studies have found that femoral stem design affected the early incidence of periprosthetic fracture. A study showed that compared with single-taper and double-taper stems, compaction-collared stems had a significantly lower risk of postoperative Vancouver B and PFF requiring operative treatment [59]. Also, stem geometry is associated with periprosthetic fracture; studies found that, compared to straight stems, there were more reports of incidence of periprosthetic fracture with use of anatomic stems [60]. Forlenza et al. suggested that patients with osteoporosis, who were bisphosphate-naive, were more likely to sustain a periprosthetic fracture after cementless TKAs relative to cemented TKA [34]. Previous studies also showed that patients with cemented stems had a significantly lower incidence of periprosthetic fracture than those with cementless stems [61]. In addition, the effects of comorbidity should also be considered for incidence of periprosthetic fracture. In this study, the pooled odds ratio indicated that the odds of periprosthetic fracture in bisphosphate users were still significantly higher than those of non-users of bisphosphonate after adjusting for comorbidities and other risk factors. However, for the pooled hazard ratios, the comorbidities factor was not taken into account when investigating the effects of the anti-resorptive agent on periprosthetic fracture. Yet, some serious chronic diseases were included in their studies. Other risk factors, such as fragility fractures, have been demonstrated in other studies, in which Ross et al. stated that there was a significantly increased risk of periprosthetic fracture and all-cause revision for patients with a history of fragility fracture [62]. The FRAX score, which incorporates the history of fragility fracture and other risk factors to determine future risk factors, was not included in this study as the basis for screening or treatment decisions in metabolic disease. Further research should consider incorporating the FRAX score to study its relation to the incidence of periprosthetic fracture.

Waiting time for elective joint operations has been a long-standing challenge in many hospital units worldwide and can reach 6 months or more [63,64]. An observational study of 282,367 patients from the Scottish arthroplasty project, taking into account all total hip and knee replacements in the National Health Service (NHS) Scotland from 1998 to 2021, showed that the mean waiting time in 2021 for total hip arthroplasty was 283.7 days and the mean waiting time for total knee arthroplasty was 316.8 days [65]. This also provides a window of opportunity for clinicians to assess and treat bone health in the presence of osteoporosis, but further randomized controlled trials are required to confirm the efficacy. A recent study showed that the mean time of a periprosthetic fracture to occur following a TKA/THA was 2.5 years [66]. Given the short duration, research on the use of more potent agents may provide useful information in the future. A bone health optimization program should also include nutritional supplements, elimination of risk factors, including alcohol intake, fall risk assessment, and exercise and education [67]. The strength of this study includes an updated pooled prevalence of osteopenia/osteoporosis before TKA/THA. Furthermore, our systematic review and meta-analysis provide a comprehensive literature review and pooled analysis on the effects of anti-resorptive agents on the risk of periprosthetic fractures, revision, and other clinical outcomes after primary TKA/THA. The limitations of this study include the use of retrospective studies and a lack of randomized controlled trials. Furthermore, there were a limited number of studies and a lack of papers using anabolic agents. Also, this review was not registered with PROSPERO. Despite our results showing that the odds of periprosthetic fracture in bisphosphonate users tend to be higher than in bisphosphonate non-users, a direct causal relationship between bisphosphonate and periprosthetic fracture could not be established due to the retrospective study design and the limited number of included studies. In addition, from the risk of bias assessment, some studies were of medium quality, which may affect the validity of the results.

The possibility of a potential publication bias could not be eliminated completely based on Egger’s test result. Also, there may be other potential confounding factors, including femoral stem design and comorbidities that were not controlled in some of the included studies, which may interfere with the results. Furthermore, whether any difference in the effects of preoperative or postoperative use of bisphosphonate exists in periprosthetic fracture has not been determined clearly.

## 5. Conclusions

In conclusion, there is a high prevalence of osteopenia and osteoporosis amongst patients undergoing total knee and total hip arthroplasty. Bone health optimization with bisphosphonates may decrease the risk of revisions, but further research with anabolic agents should be conducted, which may be the key to preventing periprosthetic fractures. Further randomized controlled trials assessing both the effects of anti-resorptive and anabolic agents on the incidence of periprosthetic fracture in patients with osteoporosis are warranted.

## Figures and Tables

**Table 1:** Quality assessment using the Newcastle–Ottawa Scale (NOS) for cohort studies.

	Selection				Comparability		Outcome			Total Score
Cohort (Author, Year)	Representativeness of Exposed Cohort	Selection of Non-Exposed Cohort	Ascertainment of Exposure to Implants	Outcome of Interest not Present at Start of Study	Cohorts on Main Factors	Cohorts on Additional Factors	Assessment of Outcome	Sufficient Follow-Up Period	Adequacy of Follow Up of Cohorts	
Khatod et al., 2015 [32]	1	1	1	1	1	0	1	1	1	8
Namba et al., 2016 [36]	1	1	1	1	1	1	1	1	1	9
Ishii et al., 2016 [23]	1	0	1	0	0	0	1	1	1	5
Ha et al., 2020 [37]	1	1	1	0	1	0	1	1	1	7
Murahashi et al., 2020 [22]	1	0	1	0	1	1	1	1	1	7
Delsmann et al., 2021 [38]	1	0	0	0	1	1	1	1	1	6
Delsmann et al., 2022 [39]	1	0	0	0	1	1	1	1	1	6
Watanabe et al., 2022 [33]	1	1	1	0	1	1	1	1	1	8
Forlenza et al., 2024 [34]	1	1	1	1	1	1	1	1	1	9
Zhao et al., 2023 [40]	1	1	1	1	1	1	1	1	1	9
Kubo et al., 2024 [41]	0	1	0	0	1	1	1	1	1	6
Shepard et al., 2024 [42]	1	0	1	1	1	1	1	1	1	8
Serino III et al., 2024 [35]	1	1	1	1	1	1	1	1	1	9

**Table 2:** Newcastle–Ottawa Scale—adapted for cross-sectional studies.

	Selection				Comparability	Outcome		Total Score
Studies (Author, Year)	Representativeness of Exposed Cohort	Sample Size	Non-Respondents	Ascertainment of the Exposure	Confounder Controlled	Assessment of Outcome	Statistical Test	
Sadigursky et al., 2017 [21]	0	0	1	1	1	1	1	7
Lingard et al., 2010 [20]	1	0	1	1	1	1	1	8

## Data Availability

Not applicable.

## Supplementary Materials

The following supporting information can be downloaded at https://www.mdpi.com/article/10.3390/jcm14248769/s1. Figure S1: Subgroup analysis by study design for the analysis of prevalence of osteopenia before TKA/THA surgery; Figure S2: Subgroup analysis by countries for the analysis of prevalence of osteopenia before TKA/THA surgery; Figure S3: Subgroup analysis by study design for the analysis of prevalence of osteoporosis before TKA/THA surgery; Figure S4: Subgroup analysis by countries for the analysis of prevalence of osteoporosis before TKA/THA surgery; Figure S5: Effects of osteoporosis (OP) treatment on change in periprosthetic BMD of medial femoral neck (Gruen zone 7) after total hip arthroplasty; Figure S6: Hazard ratio of aseptic revisions after surgery in subjects aged ≥65 years, compared bisphosphonate (BP) drug users to non-bisphosphonate drug users; Figure S7: Odds ratio of aseptic revisions in subjects with osteoporosis, compared bisphosphonate (BP) drug users to non-bisphosphonate drug users at 2 years postoperatively; Figure S8: Odds ratio of mortality after surgery in subjects with osteoporosis, compared bisphosphonate (BP) drug users to non-bisphosphonate drug users at 2 years postoperatively; Figure S9: The funnel plot for the publication bias; Table S1: Search strategy in the Pubmed, Embase and Web of Sciences database; Table S2: Characteristics of the included studies in the systematic review; Table S3: The included studies which studied the effects of anti-resorptive drugs on periprosthetic fracture and arthroplasty revisions; Table S4: Sensitivity analysis of studies included in the pooled analysis of prevalence of osteopenia before TKA/THA surgery; Table S5: Sensitivity analysis of studies included in the pooled analysis of prevalence of osteoporosis before TKA/THA surgery; File S1: PRISMA 2020 checklist [68].

## Abbreviations

The following abbreviations are used in this manuscript:

AOA	American Orthopaedic Association
BMD	Bone mineral density
CI	Confidence interval
DXA	Dual-energy X-ray absorptiometry
NOS	Newcastle–Ottawa scale
RCT	Randomized controlled studies
THA	Total hip arthroplasty
TKA	Total knee arthroplasty
WHO	World Health Organization

## References

[1] Collaborators G.B.D.O. (2023). Global, regional, and national burden of osteoarthritis, 1990–2020 and projections to 2050: A systematic analysis for the Global Burden of Disease Study 2021. Lancet Rheumatol..

[2] Belluzzi E., Olivotto E., Toso G., Cigolotti A., Pozzuoli A., Biz C., Trisolino G., Ruggieri P., Grigolo B., Ramonda R. (2019). Conditioned media from human osteoarthritic synovium induces inflammation in a synoviocyte cell line. Connect. Tissue Res..

[3] Sanchez-Lopez E., Coras R., Torres A., Lane N.E., Guma M. (2022). Synovial inflammation in osteoarthritis progression. Nat. Rev. Rheumatol..

[4] Ferguson R.J., Palmer A.J., Taylor A., Porter M.L., Malchau H., Glyn-Jones S. (2018). Hip replacement. Lancet.

[5] Price A.J., Alvand A., Troelsen A., Katz J.N., Hooper G., Gray A., Carr A., Beard D. (2018). Knee replacement. Lancet.

[6] Hamadouche M., Stern L.L. (2015). Periprosthetic fractures and complicated arthroplasties. Int. Orthop..

[7] Wong R.M.Y., Zu Y., Chau W.W., Tso C.Y., Liu W.H., Ng R.W.K., Chow S.K.H., Cheung W.H., Tang N., Ho K.K.W. (2022). High Charlson Comorbidity Index Score is associated with early fracture-related complication for internal fixation of neck of femur fractures. Sci. Rep..

[8] Bottle A., Griffiths R., White S., Wynn-Jones H., Aylin P., Moppett I., Chowdhury E., Wilson H., Davies B.M. (2020). Periprosthetic fractures: The next fragility fracture epidemic? A national observational study. BMJ Open.

[9] Team C.S. (2022). Management and outcomes of femoral periprosthetic fractures at the hip: Data from the characteristics, outcomes and management of periprosthetic fracture service evaluation (COMPOSE) cohort study. Bone Joint J..

[10] Bengoa F., Neufeld M.E., Howard L.C., Masri B.A. (2023). Periprosthetic Fractures After a Total Knee Arthroplasty. J. Am. Acad. Orthop. Surg..

[11] Harris A.B., Lantieri M.A., Agarwal A.R., Golladay G.J., Thakkar S.C. (2024). Osteoporosis and Total Knee Arthroplasty: Higher 5-Year Implant-Related Complications. J. Arthroplast..

[12] Daher M., Mekhael E., El-Othmani M.M. (2024). Total Hip Arthroplasty in Patients with Hip Osteoporosis: A Narrative Review. Hip Pelvis.

[13] Wong R.M.Y., Ko S.Y., Chau W.W., Lee L.C.Y., Chow S.K.H., Cheung W.H., Law S.W. (2021). The first reported fracture liaison service (FLS) for vertebral fractures in China: Is muscle the missing gap?. Arch. Osteoporos..

[14] Wong R.M.Y., Cheung W.H., Chow S.K.H., Ng R.W.K., Li W., Hsu A.Y., Wong K.K., Ho A.W., Choi S.H., Fang C.X. (2022). Recommendations on the post-acute management of the osteoporotic fracture—Patients with “very-high” Re-fracture risk. J. Orthop. Translat..

[15] Anderson P.A., Jeray K.J., Lane J.M., Binkley N.C. (2019). Bone Health Optimization: Beyond Own the Bone: AOA Critical Issues. J. Bone Joint Surg. Am..

[16] Liberati A., Altman D.G., Tetzlaff J., Mulrow C., Gøtzsche P.C., Ioannidis J.P., Clarke M., Devereaux P.J., Kleijnen J., Moher D. (2009). The PRISMA statement for reporting systematic reviews and meta-analyses of studies that evaluate healthcare interventions: Explanation and elaboration. BMJ.

[17] Higgins J.P., Altman D.G., Gøtzsche P.C., Jüni P., Moher D., Oxman A.D., Savovic J., Schulz K.F., Weeks L., Sterne J.A. (2011). The Cochrane Collaboration’s tool for assessing risk of bias in randomised trials. BMJ.

[18] Wells G.A., Shea B., O’Connell D., Peterson J., Welch V., Losos M., Tugwell P. (2000). The Newcastle-Ottawa Scale (NOS) for Assessing the Quality of Nonrandomised Studies in Meta-Analyses. https://www.ohri.ca/programs/clinical_epidemiology/oxford.asp.

[19] Herzog R., Álvarez-Pasquin M.J., Díaz C., Del Barrio J.L., Estrada J.M., Gil Á. (2013). Are healthcare workers’ intentions to vaccinate related to their knowledge, beliefs and attitudes? A systematic review. BMC Public Health.

[20] Lingard E.A., Mitchell S.Y., Francis R.M., Rawlings D., Peaston R., Birrell F.N., McCaskie A.W. (2010). The prevalence of osteoporosis in patients with severe hip and knee osteoarthritis awaiting joint arthroplasty. Age Ageing.

[21] Sadigursky D., Barretto L.A.J., Lobão D.M.V., Carneiro R.J.F., Colavolpe P.O. (2017). Osteoporosis in brazilian patients awaiting knee arthroplasty. Acta Ortop. Bras..

[22] Murahashi Y., Teramoto A., Jimbo S., Okada Y., Kamiya T., Imamura R., Takashima H., Watanabe K., Nagoya S., Yamashita T. (2020). Denosumab prevents periprosthetic bone mineral density loss in the tibial metaphysis in total knee arthroplasty. Knee.

[23] Ishii Y., Noguchi H., Sato J., Takayama S., Toyabe S.I. (2016). Preoperative Bone Mineral Density and Bone Turnover in Women Before Primary Knee Arthroplasty. Open Orthop. J..

[24] Wang C.J., Wang J.W., Weng L.H., Hsu C.C., Huang C.C., Chen H.S. (2003). The effect of alendronate on bone mineral density in the distal part of the femur and proximal part of the tibia after total knee arthroplasty. J. Bone Joint Surg. Am..

[25] Wang C.J., Wang J.W., Ko J.Y., Weng L.H., Huang C.C. (2006). Three-year changes in bone mineral density around the knee after a six-month course of oral alendronate following total knee arthroplasty. A prospective, randomized study. J. Bone Joint Surg. Am..

[26] Yamasaki S., Masuhara K., Yamaguchi K., Nakai T., Fuji T., Seino Y. (2007). Risedronate reduces postoperative bone resorption after cementless total hip arthroplasty. Osteoporos. Int..

[27] Iwamoto N., Inaba Y., Kobayashi N., Ishida T., Yukizawa Y., Saito T. (2011). A comparison of the effects of alendronate and alfacalcidol on bone mineral density around the femoral implant and in the lumbar spine after total hip arthroplasty. J. Bone Joint Surg. Am..

[28] Iwamoto N., Inaba Y., Kobayashi N., Yukizawa Y., Ike H., Ishida T., Saito T. (2014). The effectiveness of mono or combined osteoporosis drug therapy against bone mineral density loss around femoral implants after total hip arthroplasty. J. Bone Miner. Metab..

[29] Aro E., Moritz N., Mattila K., Aro H.T. (2018). A long-lasting bisphosphonate partially protects periprosthetic bone, but does not enhance initial stability of uncemented femoral stems: A randomized placebo-controlled trial of women undergoing total hip arthroplasty. J. Biomech..

[30] Aro H.T., Nazari-Farsani S., Vuopio M., Löyttyniemi E., Mattila K. (2019). Effect of Denosumab on Femoral Periprosthetic BMD and Early Femoral Stem Subsidence in Postmenopausal Women Undergoing Cementless Total Hip Arthroplasty. JBMR Plus.

[31] Nakura N., Hirakawa K., Takayanagi S., Mihara M. (2023). Denosumab prevented periprosthetic bone resorption better than risedronate after total hip arthroplasty. J. Bone Miner. Metab..

[32] Khatod M., Inacio M.C., Dell R.M., Bini S.A., Paxton E.W., Namba R.S. (2015). Association of Bisphosphonate Use and Risk of Revision After THA: Outcomes From a US Total Joint Replacement Registry. Clin. Orthop. Relat. Res..

[33] Watanabe N., Miyatake K., Takada R., Ogawa T., Amano Y., Jinno T., Koga H., Yoshii T., Okawa A. (2022). The prevalence and treatment of osteoporosis in patients undergoing total hip arthroplasty and the levels of biochemical markers of bone turnover. Bone Joint Res..

[34] Forlenza E.M., Serino J., Acuña A.J., Terhune E.B., Behery O.A., Della Valle C.J. (2024). Bisphosphonate Use in Patients Who Have Osteoporosis Does Not Increase the Risk of Periprosthetic Fracture Following Total Knee Arthroplasty. J. Arthroplast..

[35] Serino J., Terhune E.B., Harkin W.E., Weintraub M.T., Baim S., Della Valle C.J. (2024). Bisphosphonate Use May be Associated With an Increased Risk of Periprosthetic Hip Fracture. J. Arthroplast..

[36] Namba R.S., Inacio M.C., Cheetham T.C., Dell R.M., Paxton E.W., Khatod M.X. (2016). Lower Total Knee Arthroplasty Revision Risk Associated With Bisphosphonate Use, Even in Patients With Normal Bone Density. J. Arthroplast..

[37] Ha C.W., Park Y.B. (2020). Underestimation and undertreatment of osteoporosis in patients awaiting primary total knee arthroplasty. Arch. Orthop. Trauma. Surg..

[38] Delsmann M.M., Strahl A., Mühlenfeld M., Jandl N.M., Beil F.T., Ries C., Rolvien T. (2021). High prevalence and undertreatment of osteoporosis in elderly patients undergoing total hip arthroplasty. Osteoporos. Int..

[39] Delsmann M.M., Schmidt C., Mühlenfeld M., Jandl N.M., Boese C.K., Beil F.T., Rolvien T., Ries C. (2022). Prevalence of osteoporosis and osteopenia in elderly patients scheduled for total knee arthroplasty. Arch. Orthop. Trauma. Surg..

[40] Zhao A.Y., Agarwal A.R., Harris A.B., Cohen J.S., Golladay G.J., Thakkar S.C. (2023). The Association of Prior Fragility Fractures on 8-Year Periprosthetic Fracture Risk Following Total Hip Arthroplasty. J. Arthroplast..

[41] Kubo M., Nosaka Y., Hasegawa T., Kumagai K., Amano Y., Isoya E., Imai S. (2024). Osteoporosis should be evaluated by bone mineral density at the combination of the lumbar spine and ipsilateral femoral neck in female patients with knee osteoarthritis scheduled for knee arthroplasty: A retrospective observational study. J. Orthop. Sci..

[42] Shepard S., Bartholomew A., Houserman D., Bamberger H.B., Manocchio A.G. (2024). Assessing osteoporosis screening compliance in total joint surgery: A retrospective chart review. J. Osteopath. Med..

[43] Stihsen C., Springer B., Nemecek E., Olischar B., Kaider A., Windhager R., Kubista B. (2017). Cementless Total Hip Arthroplasty in Octogenarians. J. Arthroplast..

[44] Araki R., Asari T., Fukutoku T., Takeuchi K., Nakamura Y. (2024). Early Postoperative Outcomes of Periprosthetic Femoral Fracture in Patients Over 90 Years of Age. Cureus.

[45] Zura R., Xiong Z., Einhorn T., Watson J.T., Ostrum R.F., Prayson M.J., Della Rocca G.J., Mehta S., McKinley T., Wang Z. (2016). Epidemiology of Fracture Nonunion in 18 Human Bones. JAMA Surg..

[46] Chow S.K., Chim Y.N., Wang J.Y., Wong R.M., Choy V.M., Cheung W.H. (2020). Inflammatory response in postmenopausal osteoporotic fracture healing. Bone Joint Res..

[47] Choy M.V., Wong R.M., Li M.C., Wang B.Y., Liu X.D., Lee W., Cheng J.C., Chow S.K., Cheung W.H. (2020). Can we enhance osteoporotic metaphyseal fracture healing through enhancing ultrastructural and functional changes of osteocytes in cortical bone with low-magnitude high-frequency vibration?. FASEB J..

[48] Zhang N., Chim Y.N., Wang J., Wong R.M.Y., Chow S.K.H., Cheung W.H. (2020). Impaired Fracture Healing in Sarco-Osteoporotic Mice Can Be Rescued by Vibration Treatment Through Myostatin Suppression. J. Orthop. Res..

[49] Wong R.M.Y., Choy V.M.H., Li J., Li T.K., Chim Y.N., Li M.C.M., Cheng J.C.Y., Leung K.S., Chow S.K., Cheung W.H. (2021). Fibrinolysis as a target to enhance osteoporotic fracture healing by vibration therapy in a metaphyseal fracture model. Bone Joint Res..

[50] Gibbs V.N., McCulloch R.A., Dhiman P., McGill A., Taylor A.H., Palmer A.J.R., Kendrick B.J.L. (2020). Modifiable risk factors for mortality in revision total hip arthroplasty for periprosthetic fracture. Bone Joint J..

[51] Daher M., Mekhael E., El-Othmani M.M. (2024). Osteoporosis in the setting of knee arthroplasty: A narrative review. Arthroplasty.

[52] Lee J.K. (2025). Lee’s TRIAD-osteoporosis, fragility fracture, and bone health optimization. Arch. Osteoporos..

[53] Wong R.M.Y., Law S.W., Lee K.B., Chow S.K.H., Cheung W.H. (2019). Secondary prevention of fragility fractures: Instrumental role of a fracture liaison service to tackle the risk of imminent fracture. Hong Kong Med. J..

[54] Lee J.K., Leong J.F., Thong F.Y., Sharifudin M.A., Abbas A.A., Kamudin N.A.F., Rampal S., Yasin N.F., Loh K.W., Chan C.K. (2024). A Bone Health Optimization Framework for Malaysia: A position paper by the Malaysian Bone Health Optimization Network (MyBONe). Arch. Osteoporos..

[55] Seward M.W., Hannon C.P., Yuan B.J., Kearns A.E., Anderson P.A., Berry D.J., Abdel M.P. (2024). Systemic Osteoporosis and Osteopenia Among Periprosthetic Fractures After Total Hip Arthroplasty. J. Arthroplast..

[56] Busigó Torres R., Hong J., Kodali H., Poeran J., Stern B.Z., Hayden B.L., Chen D.D., Moucha C.S. (2025). Does Preoperative Bisphosphonate Use Impact the Risk of Periprosthetic Fracture Following Total Hip Arthroplasty?. J. Arthroplast..

[57] Jeong S., Lee J.W., Boucher H.R. (2023). The Effect of Preoperative Bisphosphonate Use on Total Hip Arthroplasty Outcomes. J. Arthroplast..

[58] Kadri A., Binkley N., Hare K.J., Anderson P.A. (2020). Bone Health Optimization in Orthopaedic Surgery. J. Bone Joint Surg. Am..

[59] Calkins T.E., Goetz D.D., Zalewski J.T., Jones C.A., Gaumer P.R., Ford M.C., Toy P.C., Crockarell J.R., Harkess J.W., Mihalko W.M. (2023). Hip Arthroplasty Femoral Stem Designs and Their Association With Early Postoperative Periprosthetic Femoral Fractures. J. Arthroplast..

[60] Cottino U., Dettoni F., Caputo G., Bonasia D.E., Rossi P., Rossi R. (2020). Incidence and pattern of periprosthetic hip fractures around the stem in different stem geometry. Int. Orthop..

[61] Megaloikonomos P.D., Nowak L., Shehata M., Sprague S., Bzovsky S., Epure L.M., De Petrillo G., Caron C., Laggis G., Huk O.L. (2025). Does Stem Design Affect the Incidence of Periprosthetic Femoral Fractures in Arthroplasty for Femoral Neck Fractures? A Secondary Analysis of the HEALTH Trial. J. Arthroplast..

[62] Ross A.J., Ross B.J., Lee O.C., Guild G.N., Sherman W.F. (2021). The Impact of Prior Fragility Fractures on Complications After Total Hip Arthroplasty: A Propensity Score-Matched Cohort Study. Arthroplast. Today.

[63] Hart D.A., Werle J., Robert J., Kania-Richmond A. (2021). Long wait times for knee and hip total joint replacement in Canada: An isolated health system problem, or a symptom of a larger problem?. Osteoarthr. Cartil. Open.

[64] Clement N.D., Wickramasinghe N.R., Bayram J.M., Hughes K., Oag E., Heinz N., Fraser E., Jefferies J.G., Dall G.F., Ballantyne A. (2022). Significant deterioration in quality of life and increased frailty in patients waiting more than six months for total hip or knee arthroplasty: A cross-sectional multicentre study. Bone Joint J..

[65] Jabbal M., Burt J., Clarke J., Moran M., Walmsley P., Jenkins P.J. (2024). Trends in incidence and average waiting time for arthroplasty from 1998–2021: An observational study of 282,367 patients from the Scottish arthroplasty project. Ann. R. Coll. Surg. Engl..

[66] Luzzi A., Lakra A., Murtaugh T., Shah R.P., Cooper H.J., Geller J.A. (2024). The Effect of Periprosthetic Fractures Following Total Hip and Knee Arthroplasty on Long-Term Functional Outcomes and Quality of Life. Arthroplast. Today.

[67] Anderson P.A., Dimar J.R., Lane J.M., Lehman R.A. (2021). Rationale for Bone Health Optimization in Patients Undergoing Orthopaedic Surgery. Instr. Course Lect..

[68] Page M.J., McKenzie J.E., Bossuyt P.M., Boutron I., Hoffmann T.C., Mulrow C.D., Shamseer L., Tetzlaff J.M., Akl E.A., Brennan S.E. (2021). The PRISMA 2020 statement: An updated guideline for reporting systematic reviews. BMJ.

