# Supplementary material for: High Prevalence of Osteopenia and Osteoporosis in Total Hip and Total Knee Arthroplasty Patients and Effects of Anti-Resorptive Agents on Bone Health Optimization: A Systematic Review and Meta-Analysis
Source: J Clin Med. 2025 Dec 11;14(24):8769. doi: 10.3390/jcm14248769 (PMC12733446; doi:10.3390/jcm14248769)
Supplement: Supplementary file 1 [file jcm-14-08769-s001.zip › jcm-3980460-supplementary.pdf]

Supplementary Information:

Supplementary Figure 1 Subgroup analysis by study design for the analysis of prevalence of osteopenia before TKA/THA surgery

Supplementary Figure 2 Subgroup analysis by countries for the analysis of prevalence of osteopenia before TKA/THA surgery

Supplementary Figure 3 Subgroup analysis by study design for the analysis of prevalence of osteoporosis before TKA/THA surgery

Supplementary Figure 4 Subgroup analysis by countries for the analysis of prevalence of osteoporosis before TKA/THA surgery

Supplementary Figure 5 Effects of osteoporosis (OP) treatment on change in periprosthetic BMD of medial femoral neck (Gruen zone 7) after total hip arthroplasty

Supplementary Figure 6 Hazard ratio of aseptic revisions after surgery in subjects aged ≥65 years, compared Bisphosphonates (BP) drug users to non-Bisphosphonates drug users

Supplementary Figure 7 Odds ratio of aseptic revisions in subjects with osteoporosis, compared Bisphosphonates (BP) drug users to non-Bisphosphonates drug users at 2 years postoperatively

Supplementary Figure 8 Odds ratio of mortality after surgery in subjects with osteoporosis, compared Bisphosphonates (BP) drug users to non-Bisphosphonates drug users at 2 years postoperatively

Supplementary Figure 9 The Funnel plot for the publication bias

Supplementary Table 1 Search strategy in the Pubmed, Embase and Web of Sciences database

Supplementary Table 2 Characteristics of the included studies in the systematic review

Supplementary Table 3 The included studies which studied the effects of antiresorptive drugs on periprosthetic fracture and arthroplasty revisions

Supplementary table 4 Sensitivity analysis of studies included in the pooled analysis of prevalence of osteopenia before TKA/THA surgery

Supplementary table 5 Sensitivity analysis of studies included in the pooled analysis of prevalence of osteoporosis before TKA/THA surgery

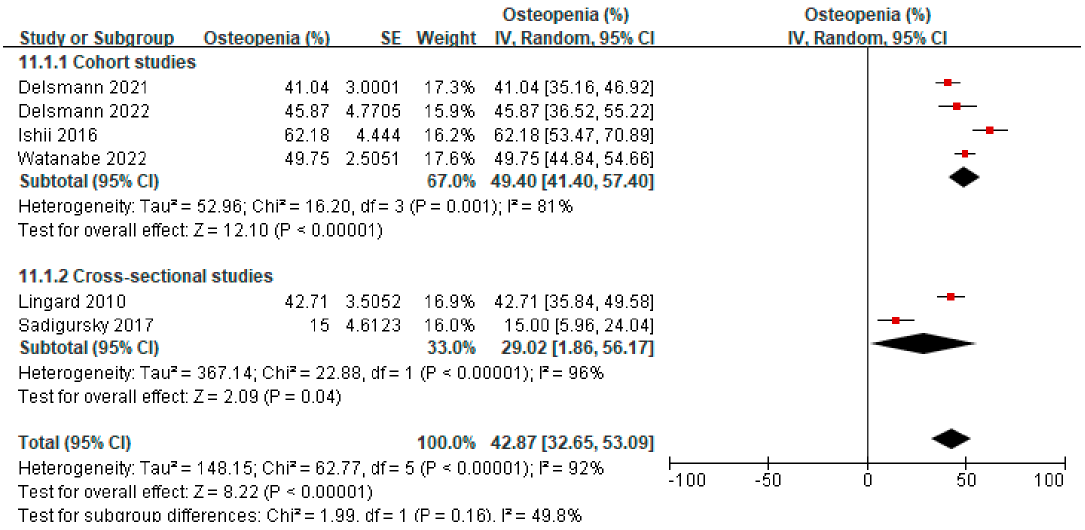

Supplementary Figure 1 Subgroup analysis by study design for the analysis of prevalence of osteopenia before TKA/THA surgery

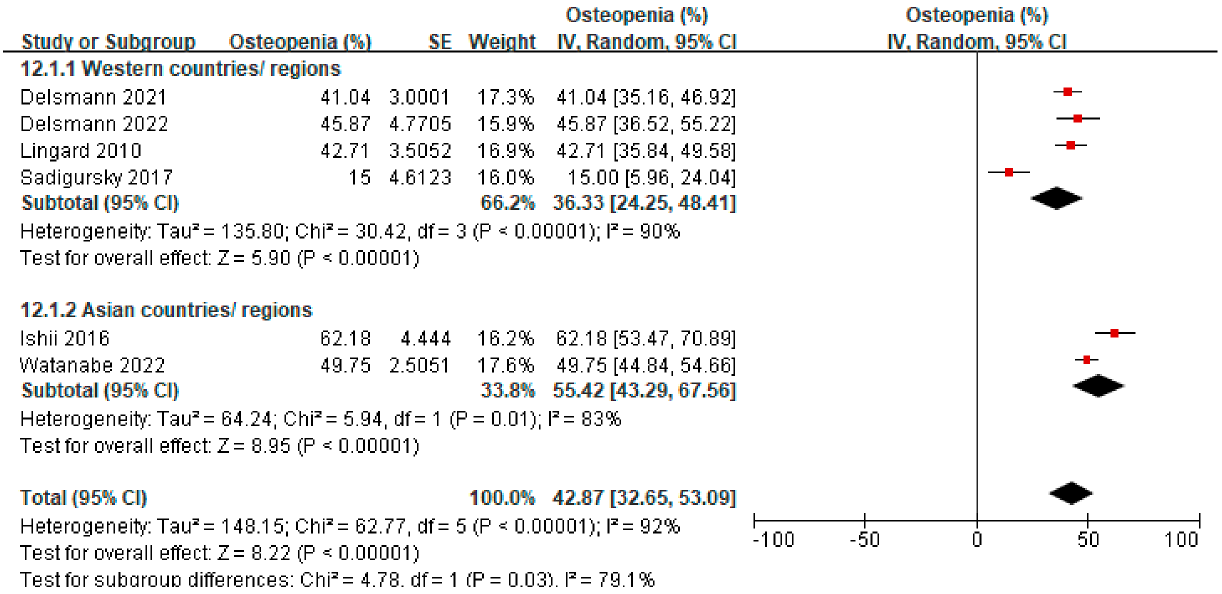

Supplementary Figure 2 Subgroup analysis by countries for the analysis of prevalence of osteopenia before TKA/THA surgery

Study or Subgroup	Osteoporosis (%)	SE	Weight	Osteoporosis (%) IV, Random, 95% CI
**13.1.1 Cohort studies**				
Delsmann 2021	18.28	2.3572	10.3%	18.28 [13.66, 22.90]
Delsmann 2022	17.43	3.6327	9.9%	17.43 [10.31, 24.55]
Ha 2020	50	1.1276	10.6%	50.00 [47.79, 52.21]
Ishii 2016	17.65	3.495	9.9%	17.65 [10.80, 24.50]
Kubo 2024	56	7.0205	8.2%	56.00 [42.24, 69.76]
Namba 2016	16.05	0.2806	10.6%	16.05 [15.50, 16.60]
Shepard 2024	28.4	1.9745	10.4%	28.40 [24.53, 32.27]
Zhao 2023	1.85	0.0204	10.6%	1.85 [1.81, 1.89]
**Subtotal (95% CI)**			**80.5%**	**24.95 [15.74, 34.15]**
Heterogeneity: Tau² = 166.93; Chi² = 4689.88, df = 7 (P < 0.00001); I² = 100%				
Test for overall effect: Z = 5.31 (P < 0.00001)				
**13.1.2 Cross-sectional studies**				
Lingard 2010	23.12	2.9899	10.1%	23.12 [17.26, 28.98]
Sadigursky 2017	16.67	4.8113	9.3%	16.67 [7.24, 26.10]
**Subtotal (95% CI)**			**19.5%**	**21.00 [15.06, 26.94]**
Heterogeneity: Tau² = 4.76; Chi² = 1.30, df = 1 (P = 0.25); I² = 23%				
Test for overall effect: Z = 6.93 (P < 0.00001)				
**Total (95% CI)**			**100.0%**	**23.99 [15.72, 32.26]**
Heterogeneity: Tau² = 167.14; Chi² = 4749.40, df = 9 (P < 0.00001); I² = 100%				
Test for overall effect: Z = 5.69 (P < 0.00001)				
Test for subgroup differences: Chi² = 0.50. df = 1 (P = 0.48). I² = 0%				

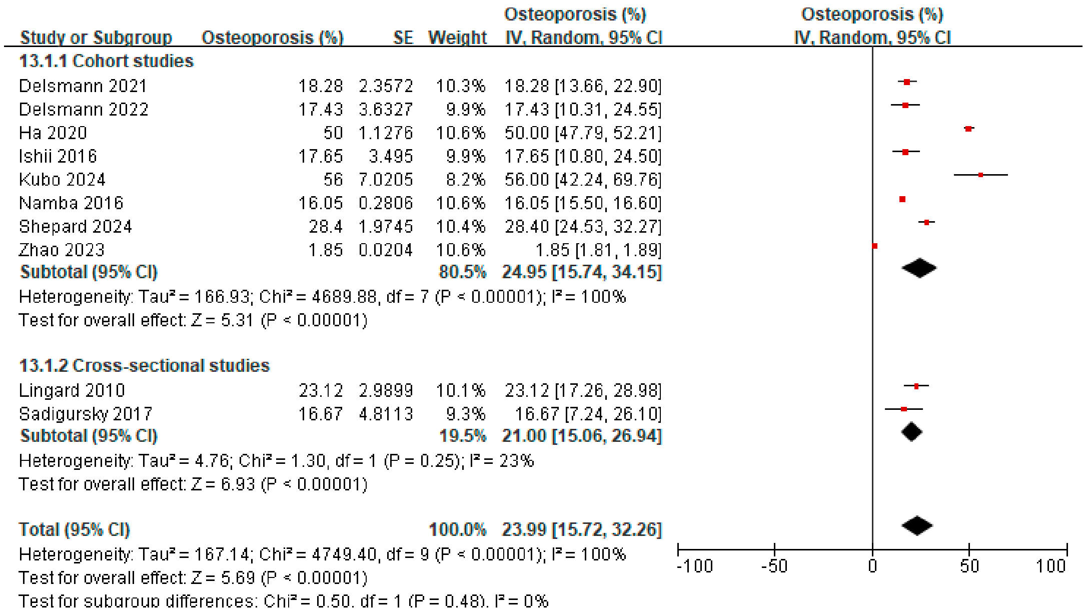

Supplementary Figure 3 Subgroup analysis by study design for the analysis of prevalence of osteoporosis before TKA/THA surgery

Study or Subgroup	Osteoporosis (%)	SE	Weight	Osteoporosis (%) IV, Random, 95% CI
**14.1.1 Western countries/ regions**				
Delsmann 2021	18.28	2.3572	10.3%	18.28 [13.66, 22.90]
Delsmann 2022	17.43	3.6327	9.9%	17.43 [10.31, 24.55]
Lingard 2010	23.12	2.9899	10.1%	23.12 [17.26, 28.98]
Namba 2016	16.05	0.2806	10.6%	16.05 [15.50, 16.60]
Sadigursky 2017	16.67	4.8113	9.3%	16.67 [7.24, 26.10]
Shepard 2024	28.4	1.9745	10.4%	28.40 [24.53, 32.27]
Zhao 2023	1.85	0.0204	10.6%	1.85 [1.81, 1.89]
**Subtotal (95% CI)**			**71.3%**	**17.29 [9.36, 25.21]**
Heterogeneity: Tau² = 107.06; Chi² = 2853.20, df = 6 (P < 0.00001); I² = 100%				
Test for overall effect: Z = 4.28 (P < 0.0001)				
**14.1.2 Asian countries/ regions**				
Ha 2020	50	1.1276	10.6%	50.00 [47.79, 52.21]
Ishii 2016	17.65	3.495	9.9%	17.65 [10.80, 24.50]
Kubo 2024	56	7.0205	8.2%	56.00 [42.24, 69.76]
**Subtotal (95% CI)**			**28.7%**	**40.90 [17.20, 64.59]**
Heterogeneity: Tau² = 418.40; Chi² = 79.22, df = 2 (P < 0.00001); I² = 97%				
Test for overall effect: Z = 3.38 (P = 0.0007)				
**Total (95% CI)**			**100.0%**	**23.99 [15.72, 32.26]**
Heterogeneity: Tau² = 167.14; Chi² = 4749.40, df = 9 (P < 0.00001); I² = 100%				
Test for overall effect: Z = 5.69 (P < 0.00001)				
Test for subgroup differences: Chi² = 3.43. df = 1 (P = 0.06). I² = 70.9%				

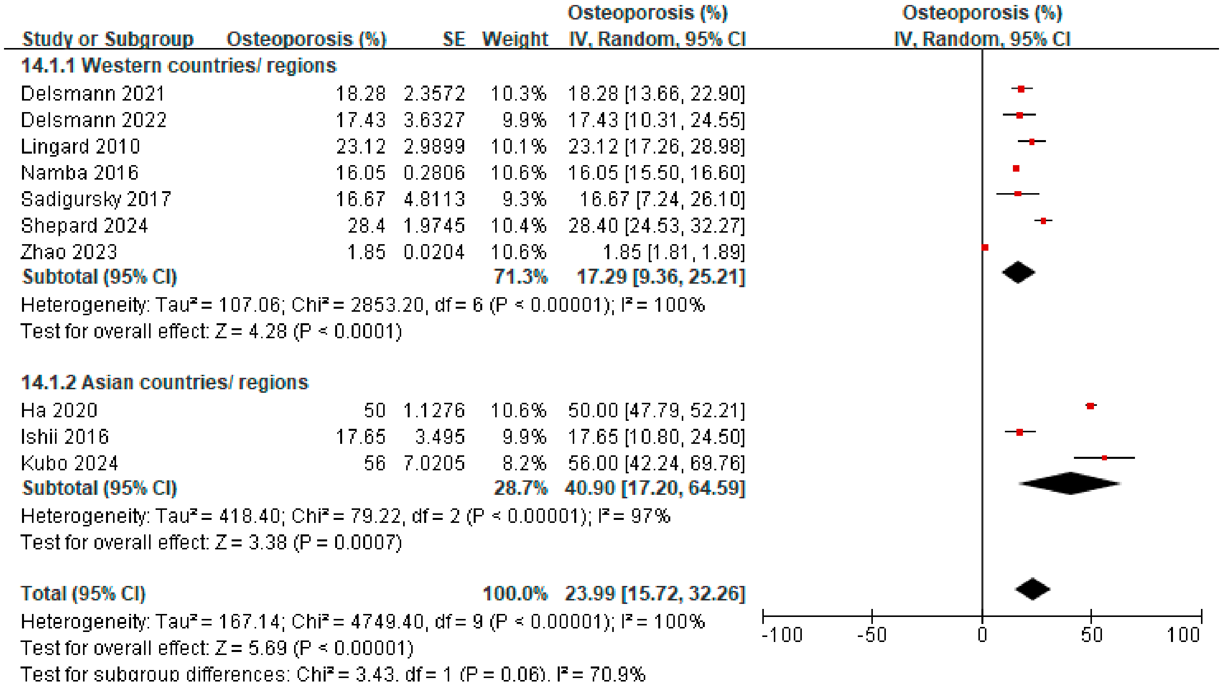

Supplementary Figure 4 Subgroup analysis by countries for the analysis of prevalence of osteoporosis before TKA/THA surgery

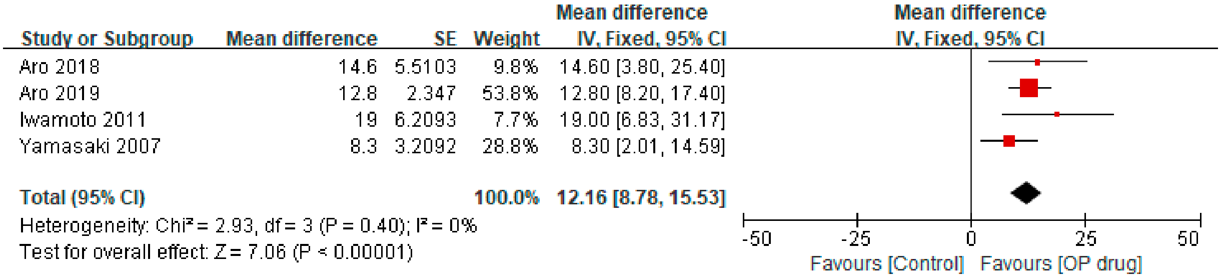

Supplementary Figure 5 Effects of osteoporosis (OP) treatment on change (%) in periprosthetic BMD of medial femoral neck (Gruen zone 7) after total hip arthroplasty

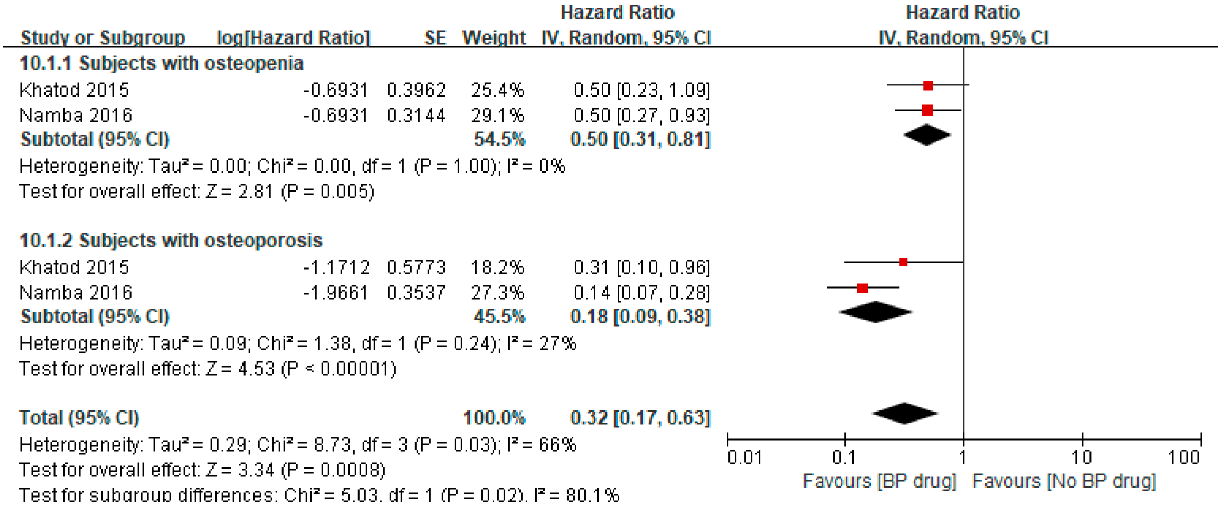

Supplementary Figure 6 Hazard ratio of aseptic revisions after surgery in subjects aged ≥65 years, compared Bisphosphonates (BP) drug users to non-Bisphosphonates drug users

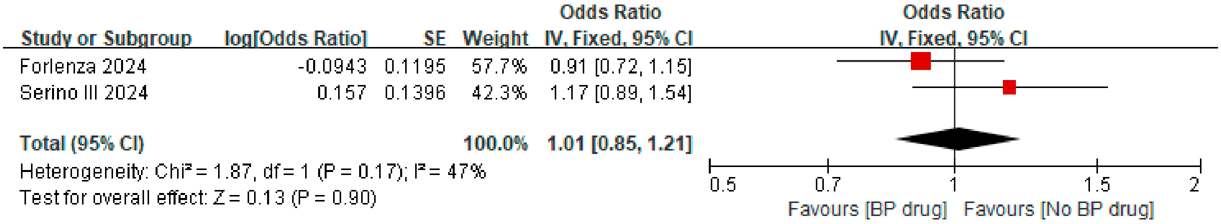

Supplementary Figure 7 Odds ratio of aseptic revisions in subjects with osteoporosis, compared Bisphosphonates (BP) drug users to non-Bisphosphonates drug users at 2 years postoperatively

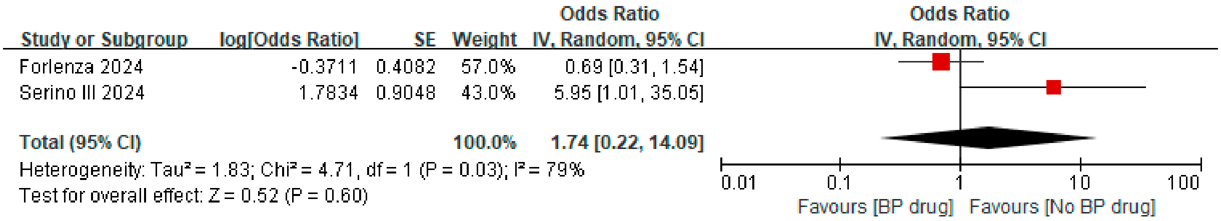

Supplementary Figure 8 Odds ratio of mortality after surgery in subjects with osteoporosis, compared Bisphosphonates (BP) drug users to non-Bisphosphonates drug users at 2 years postoperatively

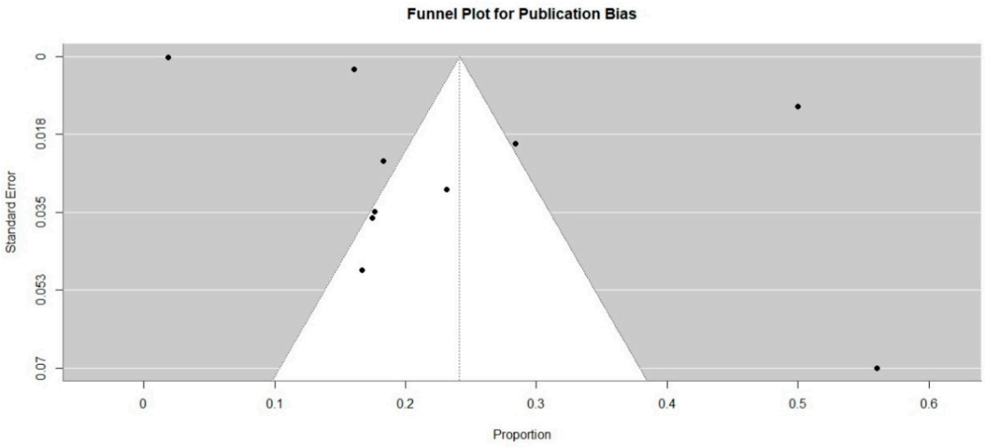

Supplementary Figure 9 The Funnel plot for the publication bias

Supplementary Table 1 Search strategy in the Pubmed, Embase and Web of Sciences databases

	Search Strategy
Search 1	“Revision” [All Field]
Search 2	“Periprosthetic fracture” [All Field]
Search 3	(Search 1 AND Search 2) [All Field]
Search 4	“Osteop*” [All Field]
Search 5	“Total Knee*” [All Field]
Search 6	“Total hip*” [All Field]
Search 7	(Search 5 AND Search 6) [All Field]
Final search	(Search 3 AND Search 4 AND Search 7)

Supplementary Table 2 Characteristics of the included studies in the systematic review

Author, year	Country /region	Study design	Surgical indication	Type of surgery	Sample size	Age (years) (Mean±SD)	Gender (Male/Female)	Percentage of osteopenia/osteoporosis
**Prevalence of Osteopenia/Osteoporosis**								
Lingard et al, 2010 [1]	UK	Cross-sectional	Osteoarthritis (OA)	TKA, THA	199	72±4 (range: 65-80)	86/113	**Osteoporosis:** At any site: 46 (23%), LS: 17 (9%), Forearm: 27 (14%), Index proximal femur: 13 (8%), Contralateral proximal femur: 9 (6%)<br>**Osteopenia:** At any sites: 85 (43%), LS: 48 (24%), Forearm: 82 (42%), Index proximal femur: 58 (36%), Contralateral proximal femur: 48 (31%)<br>**Normal:** 68 (34%)
Sadigursky et al, 2017 [2]	Brazil	Cross-sectional	OA	TKA	60	71.4±6.9 (range:58-85)	All females	**Osteoporosis:** 10 (16.7%)<br>**Osteopenia:** 9 (15%)<br>**Normal:** 41 (68.3%)
Ishii et al, 2016 [3]	Japan	Prospective	OA	TKA	107 (119 knees)	72±7 (range:55-88)	All females	**Osteoporosis:** LS / hip: 21 (18%); LS: 14 (12%); Hip: 12 (10%)<br>**Osteopenia:** LS / hip: 74 (62%); LS: 44 (37%); Hip: 58 (49%)
Watanabe et al, 2022 [4]	Japan	Retrospective	DDH-OA, primary OA, osteonecrosis	THA	398	65.1 ± 11.6	68/330	**Osteoporosis:** Hip: 35 (8.7%), Spine: 18 (4.5%), Hip/Spine: 46 (11.6%)<br>**Osteopenia:** Hip**:** 184 (46.2%), Spine:

			of femoral head, rapidly destructive coxarthrosis, RA					115 (28.9), Hip/Spine: 198 (49.7) **Osteopenia/ osteoporosis:** Hip: 219 (55.0%), Spine: 133 (33.4%), Hip/Spine: 244 (61.3%)
Ha et al, 2020 [5]	South Korea	Retrospective	Advanced knee OA	TKA	1972	67.9 (range:50-89)	143/1829	**Osteoporosis:** 986 (50.0%)
Delsmann et al, 2021 [6]	Germany	Retrospective	End-stage OA	TKA	268	Female: 78.6±4.7 Male: 77.6±5.0 (Aged≥70)	90/178	**Osteoporosis:** 49 (18%) **Osteopenia:** 110 (41%)
Delsmann et al, 2022 [7]	Germany	Retrospective	End-stage OA	TKA	109	Female: 78.0±4.1 Male: 77.7±4.3 (Aged≥70)	37/72	**Osteoporosis:** 19 (17.4%) **Osteopenia:** 50 (45.9%)
Zhao et al, 2023 [8]	USA	Retrospective	OA	THA	427282	With prior FF: 69.88 No prior FF: 66.98 (Aged >50)	241,824/ 185,458	**Osteoporosis:** 7888/ 427,282 (1.85%)

Kubo et al, 2024 [9]	Japan	Retrospective	Knee OA	TKA	50	75.3±8.2 (range:56-91)	All females	**Osteoporosis:** At LS: 14 (28%), Contralateral total hip: 5 (10%), Ipsilateral total hip: 5 (10%), Contralateral femoral neck: 11 (22%), Ipsilateral femoral neck: 15 (30%), LS+ Contralateral/ Ipsilateral total hip: 16 (32%), LS+ Ipsilateral femoral neck: 32%<br>In any of the 5 sites: 28 (56%)
Shepard et al, 2024 [10]	USA	Retrospective	N/A	Elective total joint surgery	2160	74 (range:65-106)	835/ 1325	With DXA scan: 521 (24.1%)<br>**Osteoporosis:** 148/521 (28.4%) (67 due to insufficiency fractures, 81 diagnosed by DXA)
**Effects of antiresorptive**								
Murahashi et al, 2020 [11]	Japan	Prospective	Knee OA	Cemented TKA	N=28<br>A: 13<br>B: 15	A: 76.9±7.3<br>B: 75.3±8.7	2/26<br>A: 1/12<br>B: 1/14	N/A (included subjects with osteoporosis only)
Wang et al, 2003 [12]	Taiwan	RCT	OA, rheumatoid arthritis (RA), osteonecrosis	Cemented TKA	N=69<br>A: 48<br>B: 48	A: 70.0±7.0<br>B: 71.0±7.0	All females	N/A

Wang et al, 2006 [13]	Taiwan	RCT	Knee OA	Cemented TKA	N=54<br>A: 29<br>B: 25	A: 69.8±5.9<br>B: 69.7±6.7	All females	N/A
Yamasaki et al, 2007 [14]	Japan	RCT	OA secondary to acetabular dysplasia	Uncemented THA	N=41<br>A: 19<br>B: 21	A: 66.8±6.5<br>B: 66.7±4.4	A: 2/17<br>B: 2/19	N/A
Iwamoto et al, 2011 [15]	Japan	RCT	OA	Uncemented THA	N=60<br>A: 18<br>B: 14<br>C: 22	65 ± 10	14/46	N/A
Iwamoto et al, 2014 [16]	Japan	RCT	Hemi-OA	Uncemented THA	A: 18<br>B: 20<br>C: 22	A: 64±10<br>B: 65±8<br>C: 65±9	51/13	N/A
Aro et al, 2018 [17]	Finland	RCT	Advanced degenerative hip OA	Uncemented THA	N=49<br>A: 25<br>B: 24	A: 65.3±8.0<br>B: 71.0±9.5<br>(range:51-85)	All females	In proximal femur:<br>Osteopenia: A: 8 (32%), B: 8 (36%)<br>Osteoporosis: A: 1 (4%), B: 0 (0%)<br>In vertebrae:<br>Osteopenia: A: 9 (36%), B: 3 (12.5%)<br>Osteoporosis: A: 2 (8%), B: 3 (12.5%)<br>In distal radius:<br>Osteopenia: A: 11 (44%), B: 8 (33%)<br>Osteoporosis: A: 3 (12%), B: 7 (29%)

Aro et al, 2019 [18]	Finland	RCT	Hip OA	Uncemented THA	N=65<br>A: 33<br>B: 32	A: 69.1±5.2<br>B: 69.1±5.9	All females	At proximal femur of both hips & LS:<br>Normal: A: 16 (48%), B: 15 (47%)<br>Osteopenia: A: 15 (48%), B: 17 (53%)<br>Osteoporosis: A: 2 (6%), B: 0 (0%)
Nakura et al, 2023 [19]	Japan	RCT	Hip OA	Uncemented THA	N=82<br>A: 40<br>B: 42	A: 69.9±5.00<br>B: 68.3±6.32	5/77<br>A: 1/39<br>B: 4/38	Osteoporosis<br>A: 32%<br>B: 35%
Khatod et al, 2015 [20]	USA	Retrospective	OA	Elective THA	N=7510<br>A: 1891<br>B: 5619	N/A (for aged≥50 only)	N/A (for aged≥50 only)	N/A (for aged≥50 only)
Namba et al, 2016 [21]	USA	Retrospective	OA	Uncemented / cemented TKA	20784	N/A (for aged≥50 only)	N/A (for aged≥50 only)	**Osteopenia:** 8049 (47.5%)<br>**Osteoporosis:** 2720 (16.1%)<br>**Normal:** 6178 (36.5%)<br>**No scan:** 3837 (18.5%)<br>(DXA measured within 5 years of TKA)
Forlenza et al, 2024 [22]	USA	Retrospective	N/A	Uncemented / cemented elective TKA	N=42116<br>A: 21058<br>B: 21058	A: 71±6.4<br>B: 71±6.4	A: 912/ 20146<br>B: 912/ 20146	N/A (included subjects with osteoporosis only)

Serino III et al, 2024 [23]	USA	Retrospective	N/A	Elective THA	N=19688<br>A: 9844<br>B: 9844	A: 70.9±6.7<br>B: 70.9±6.7	A: 554/ 9290<br>B: 554/ 9290	N/A (included subjects with osteoporosis only)

TKA, total knee arthroplasty; THA, total hip arthroplasty; LS, lumbar spine; FF, fragility fracture; DDH, Developmental Dysplasia of the Hip; OA, osteoarthritis; DXA, dual-energy x-ray absorptiometry, RA, rheumatoid arthritis.

Supplementary Table 3 The included studies which studied the effects of antiresorptive drugs on periprosthetic fracture and arthroplasty revisions

Author, year	Study design	Use of osteoporosis drug	Time of DXA scan	Periprosthetic fracture	Arthroplasty revision	Other clinical outcomes after antiresorptive drug treatment
Murahashi et al, 2020 [11]	Prospective	Started day 1 post-op for 12 months<br><br>A: Denosumab, 60 mg every six months + 0.5 µg vitamin D3 daily<br>B: 0.5 µg active vitamin D3 daily	1st week, 3, 6, 12 months post-op	A: 0<br>B: 0	N/A	**% change in total femur BMD & periprosthetic tibial bone at month 12**<br>Total femur: A (1.52±3.39), B (−0.90±4.51), p=0.180<br>M1: A (−0.72±10.17), B (−19.65±14.16), p=0.002<br>M2: A (5.95±10.25), B (−9.94±12.10), p=0.003<br>M3: A (4.35±7.17), B (−3.80±10.81), p=0.052<br>L1: A (9.04±14.65), B (7.82±24.35), p=0.889<br>L2: A (12.14±12.60), B (2.19±14.98), p=0.108<br>L3: A (2.78±7.71), B (−2.10±12.70), p=0.292<br>Distal tibia: A (2.04±6.36), B (−4.40±11.95), p=0.135
Wang et al, 2003 [12]	RCT	Started 2nd postoperative (post-op) day for 6 months	Pre-op & 6, 12 months post-op	A: 0<br>B: 0	N/A	**BMD in distal part of femur**<br>6 months post-op: A (+10.0% ± 22.2%), B (−13.8% ± 17.4%), p<0.001

		A: Alendronate, oral 10 mg/day<br>B: Control, no alendronate				12 months post-op: A (+1.9% ± 14.9%), B (−7.8% ± 20.9%), p=0.033<br>**BMD in proximal part of tibia**<br>6 months post-op: A (+9.4% ± 10.2%), B (−6.5% ± 11.2%), p<0.001<br>12 months post-op: A (+5.4% ± 12.7%), B (−3.6% ± 15.4%), p=0.011
Wang et al, 2006 [13]	RCT	Started 2nd post-op day for 6 months<br>A: Alendronate, oral 10 mg/day<br>B: Control, no alendronate	6, 12, 36 months after TKA	A: 0<br>B: 0	N/A	**BMD in proximal part of tibia**<br>12 months post-op: A (+6.2% ± 14.1%), B: (–6.1% ± 13.0%), p<0.01<br>3 years post-op: A (–2.9% ± 8.8%), B (–8.3% ± 10.6%), p=0.10<br>**Change of BMD in region of interest (ROI) in proximal part of tibia at 3 years post-op**<br>R1: A (–12.5% ± 44.7%), B (–10.3% ± 16.6%), p=0.41<br>R2: A (+2.6% ± 59.0%), B (+0.2% ± 24.4%), p=0.43<br>R3: A (–8.1% ± 21.7%), B (–7.5% ± 23.9%), p=0.46

Yamasaki et al, 2007 [14]	RCT	Started 1 week post-op for 6 months<br><br>A: Risedronate, oral 2.5 mg/day<br>B: Calcium lactate, oral 3 g/day	3 weeks, 6 months post-op	A: 0<br>B: 0	N/A	**BMD ratio**<br>Zone 7: A (88.1±10.0), B (79.8±10.3)
Iwamoto et al, 2011 [15]	RCT	Started day 1 post-op to week 48<br><br>A: Alendronate, oral 5 mg/day<br>B: Alfacalcidol, oral 1 mg/day<br>C: No medication	1 week, 12, 24, 48 weeks post-op	N/A	A: 0<br>B: 0	**Mean Periprosthetic BMD at Region 7**<br>24 weeks post-op: A (92% ± 15%), B (73% ± 11%), C (78% ± 18%)<br>48 weeks post-op: A (92% ± 22%), B (73% ± 13%), C (73% ± 16%)<br>**Mean BMD at LS at 48 weeks post-op**<br>A (103% ± 10%), B (103% ± 18%), C (92% ± 10%)
Iwamoto et al, 2014 [16]	RCT	Started day 1 post-op to week 48<br><br>A: Alendronate alone, oral 5 mg/day<br>B: Alendronate, oral 5 mg/day + Alfacalcidol, oral 1 ug/day<br>C: No medication	1 week, 12, 24, 48 weeks post-op	N/A	N/A	**Mean Periprosthetic BMD at Region 7**<br>24 weeks post-op: A (92 ± 15%), B (92 ± 13%), C (78 ± 18)<br>48 weeks post-op: A (92 ± 22%), B (91 ± 17), C (73 ± 16 %), p<0.0001

Aro et al, 2018 [17]	RCT	Started before discharge from hospital for 1 year<br><br>A: A single infusion of 5mg Zoledronic acid<br>B: Placebo	3 days, 3, 6, 12 months and 4 years post-op	A: 0<br>B: 1 (at 9 years post-surgery)	A: 0<br>B: 0	**Periprosthetic BMD at 4 years post-op**<br>At zone 7: 14.6% (95% CI: 3.8-25.3) higher in A than B<br>At zone 1: A: -16.9% (95% CI: 21.9-11.9), B: 22.5% (95% CI: 30.6-14.4)<br>**Femoral stem migration**<br>A had no significant effect (p=0.79)<br>**Systemic BMD**<br>A: +2.2% (95% CI: 0.2-4.3%) in vertebral BMD significantly but not in B
Aro et al, 2019 [18]	RCT	Started 1 month pre-op for 12 months:<br><br>A: Denosumab, 60 mg subcutaneous injections, once every 6 months<br>B: Placebo once every 6 months	Within 4 days post-op and at 12, 22, 48 weeks post-op	A: 0<br>B: 0	A: 0<br>B: 0	**% change in periprosthetic BMD of proximal femur at week 48 post-op**<br>Zone 7: A (-5.3%), B (-18.1%) (difference: 12.8%, 95% CI: 8.2–17.4; p<0.001)<br>Compared with B (p<0.001 for all), in A:<br>Zone 1 efficacy: +12.8% (95% CI: 6.9-18.6)<br>Zone 6 efficacy: +7.9% (95%: CI 3.5-12.3)<br>Zone 1-7 efficacy: +5.5% (95% CI: 3.1-7.9)<br>**Periprosthetic BMD of zone 7 at 3 years post-op compared with baseline**

						A: –12.6% (95% CI: –18.5 to–6.6), B: –17.3% (95% CI:–21.8 to–12.8)
Nakura et al, 2023 [19]	RCT	A: Denosumab, 60 mg subcutaneous injection (Started day 1 post-op and every 6 months for 2 years)<br><br>B: Risedronate, oral 17.5 mg (Started day 3 post-op and weekly thereafter)	Baseline (day 5 post-op) and 6, 12, 18, 24 months post-op	N/A	N/A	**Mean BMD scores in femoral stem**<br>Zones 1, 2, 6, and 7 significantly higher in Denosumab group than in Risedronate group at 6, 12, 18, and 24 months post-op<br>**Mean % change in BMD score from baseline to 24 months post-op**<br>Zone 1: A (+11.9%), B (−9.6%)<br>Zone 2: A (+2.9%), B (–3.6%)<br>Zone 3: A (+3.3%), B (−1.8%)<br>Zone 4: A (−0.7%), B (−4.3%)<br>Zone 6: A (+8.1%), B (−2.3%)<br>Zone 7: A (+5.9%), B (−19.2%)
Khatod et al, 2015 [20]	Retrospective	BP users were defined as high adherence and use for >= 6 months at any time during study period<br><br>BP included risedronate sodium, alendronate sodium, ibandronate	Within 5 years of primary THA	**In aged group≥ 65: Periprosthetic fracture (ref: BP nonusers)**<br>**Osteopenia**: HR=0.78, 95% CI: 0.32-1.88, p=0.576<br>**Osteoporosis:**	**In aged group≥ 65: All-cause revisions (ref: BP nonusers)**<br>Osteopenia: HR=0.51, 95% CI: 0.27-0.95, p=0.035<br>Osteoporosis: HR=0.24, 95% CI: 0.08-0.76, p=0.016	N/A

		sodium, alendronate-sodium-cholecalciferol, etidronate disodium, and zoledronic acid were identified		HR=1.23, 95% CI: 0.35-4.37, p=0.744<br>**Normal:** HR=2.04, 95% CI: 0.21-19.4, p=0.536<br>**Overall** (aged >=65 years): HR=1.65, 95% CI: 0.98-2.78, p=0.061<br>(Period: 2005-2010, mean follow-up: 2.5±1.7 years)	Normal: HR=0.56, 95% CI: 0.17-1.78, p=0.325<br>**Aseptic revisions (ref: BP nonusers)**<br>Osteopenia: HR=0.50, 95% CI: 0.23-1.06, p=0.071<br>Osteoporosis: HR=0.31, 95% CI: 0.10-1.00, p=0.051<br>(Period: 2001-2010)	
Namba et al, 2016 [21]	Retrospective	BP users were defined as ≥2 medication prescriptions with no revision or any medication ≥6 months before revision, n=15,234 (73.3%)<br><br>BP nonusers, n=5550 (26.7%)<br><br>BP included risedronate sodium, alendronate sodium, ibandronate	Within 5 years of primary TKA	**In aged group≥ 65:**<br>**Normal**: HR=1.59 (95% CI: 0.18-14.24), p=0.677<br>**Osteopenia:** HR=2.17 (95% CI: 0.76-6.17), p=0.148<br>**Osteoporosis:** HR=1.60 (95% CI: 0.43-6.00), p=0.483<br>(Period: 2005-2011, mean follow-up: 2.8±2 years)	**In aged group≥ 65:**<br>**All-cause revisions (ref: BP nonusers)**<br>Normal: HR 0.12, 95% CI: 0.02-0.87, p=0.036<br>Osteopenia: HR 0.32, 95% CI: 0.19-0.53, p<0.001<br>Osteoporosis: HR 0.11, 95% CI: 0.06-0.18, p=NA<br>**Aseptic revisions (ref: BP nonusers)**<br>Osteopenia: HR=0.50,	N/A

		sodium, alendronate-sodium-cholecalciferol, etidronate disodium, and zoledronic acid were identified			95% CI: 0.27-0.90, p=0.020<br>Osteoporosis: HR=0.14, 95% CI: 0.07-0.31, p<0.001<br>(Period: 2001-2011)	
Forlenza et al, 2024 [22]	Retrospective	A: Bisphosphonate (BP) users: continuous BP prescription for ≥ 6 months pre-op<br><br>B: BP-naïve had not been prescribed BP at any point either pre or post-op	N/A	At 2-year after TKA: Overall OR =1.24, 95% CI: 0.99-1.56, p=0.060 (ref: BP nonusers)	At 2-year after TKA: Overall OR=0.84, 95% CI: 0.72-0.97, p=0.021 (ref: BP nonusers)	N/A
Serino III et al, 2024 [23]	Retrospective	BP users: continuous prescription for BP for a minimum of 6 months preoperatively<br><br>A: BP users<br>B: BP naïve	N/A	At 2-year after THA:<br>A: 188 (1.9%),<br>B: 146 (1.5%),<br>p=0.024<br><br>OR=1.29, 95% CI: 1.04-1.61, p=0.022 (ref: BP naïve)	At 2-year after THA:<br>A: 282 (2.9%),<br>B: 239 (2.4%), p=0.062<br><br>OR=1.19, 95% CI: 1.00-1.41, p=0.056 (ref: BP naïve)	**Periprosthetic Joint Infection**<br>A: 156 (1.6%)<br>B: 170 (1.7%), p=0.468<br>OR=0.92, 95% CI: 0.73-1.14, p=0.435 (ref: BP naïve)<br>**Aseptic Loosening**<br>A: 112 (1.1%)<br>B: 96 (1.0%), p=0.296<br>OR=1.17, 95% CI: 0.89-1.54, p=0.261

			(ref: BP naïve)<br>**Dislocation**<br>A: 156 (1.6%)<br>B: 169 (1.7%), p=0.502<br>OR=0.92, 95% CI: 0.74-1.15, p=0.467<br>(ref: BP naïve)<br>**Mortality**<br>A:6 (0.1%)<br>B: 1 (0.0%), p=0.131<br>OR=5.95, 95% CI: 1.01-112.38, p=0.099<br>(ref: BP naïve)

Pre-op, preoperative; post-op, postoperative; BMD, bone mineral density; TKA, total knee arthroplasty; THA, total hip arthroplasty; ROI, region of interest; BP, bisphosphonate; HR, hazard ratio; OR, odds ratio; CI, confidence interval; FF, fragility fracture; OP, osteoporosis; SD, standard deviation.

Supplementary table 4 Sensitivity analysis of studies included in the pooled analysis of prevalence of osteopenia before TKA/THA surgery

	No. of studies	Effect estimates		Heterogeneity	
		**% (95% CI)**	**P value**	**I^2 (%)**	**P value**
**Prevalence of osteopenia**					
-high quality study only	3	36.16 (17.87, 54.45)	0.00001	95	<0.00001
-Without Delsmann 2021	5	43.21 (30.21, 56.22)	<0.00001	93	<0.00001
-Without Delsmann 2022	5	42.28 (30.33, 54.23)	<0.00001	94	<0.00001
-Without Ishii 2016	5	39.19 (28.97, 49.41)	<0.00001	91	<0.00001
-Without Lingard 2010	5	42.87 (30.23, 55.52)	<0.00001	94	<0.00001
-Without Sadigursky 2017	5	47.99 (41.44, 54.54)	<0.00001	78	0.001
-Without Watanabe 2022	5	41.39 (28.46, 54.32)	<0.00001	93	<0.00001

CI, confidence interval.

Supplementary table 5 Sensitivity analysis of studies included in the pooled analysis of prevalence of osteoporosis before TKA/THA surgery

	**No. of studies**	**Effect estimates**		**Heterogeneity**	
		**% (95% CI)**	**P value**	I^2 **(%)**	**P value**
**Prevalence of osteoporosis**					
-high quality study only	6	22.75 (12.25, 33.25)	<0.0001	100	<0.00001
-Without Delsmann 2021	9	24.65 (15.91, 33.39)	<0.00001	100	<0.00001
-Without Delsmann 2022	9	24.71 (15.99, 33.42)	<0.00001	100	<0.00001
-Without Ha 2020	9	20.60 (13.43, 27.78)	<0.00001	100	<0.00001
-Without Ishii 2016	9	24.69 (15.97, 33.41)	<0.00001	100	<0.00001
-Without Kubo 2024	9	21.12 (12.54, 29.71)	<0.00001	100	<0.00001
-Without Lingard 2010	9	24.09 (15.38, 32.79)	<0.00001	100	<0.00001
-Without Namba 2016	9	25.33 (7.79, 42.87)	0.005	100	<0.00001
-Without Sadigursky 2017	9	24.75 (16.06, 33.43)	<0.00001	100	<0.00001
-Without Shepard 2024	9	23.47 (14.82, 32.12)	<0.00001	100	<0.00001
-Without Zhao 2023	9	26.80 (15.01, 38.58)	<0.00001	99	<0.00001

CI, confidence interval.

References:

1. Lingard EA, Mitchell SY, Francis RM, Rawlings D, Peaston R, Birrell FN, et al. The prevalence of osteoporosis in patients with severe hip and knee osteoarthritis awaiting joint arthroplasty. Age Ageing. 2010;39(2):234-9.

2. Sadigursky D, Barretto LAJ, Lobão DMV, Carneiro RJF, Colavolpe PO. Osteoporosis in brazilian patients awaiting knee arthroplasty. Acta Ortop Bras. 2017;25(3):74-7.

3. Ishii Y, Noguchi H, Sato J, Takayama S, Toyabe SI. Preoperative Bone Mineral Density and Bone Turnover in Women Before Primary Knee Arthroplasty. Open Orthop J. 2016;10:382-8.

4. Watanabe N, Miyatake K, Takada R, Ogawa T, Amano Y, Jinno T, et al. The prevalence and treatment of osteoporosis in patients undergoing total hip arthroplasty and the levels of biochemical markers of bone turnover. Bone Joint Res. 2022;11(12):873-80.

5. Ha CW, Park YB. Underestimation and undertreatment of osteoporosis in patients awaiting primary total knee arthroplasty. Arch Orthop Trauma Surg. 2020;140(8):1109-14.

6. Delsmann MM, Strahl A, Mühlenfeld M, Jandl NM, Beil FT, Ries C, et al. High prevalence and undertreatment of osteoporosis in elderly patients undergoing total hip arthroplasty. Osteoporos Int. 2021;32(8):1661-8.

7. Delsmann MM, Schmidt C, Mühlenfeld M, Jandl NM, Boese CK, Beil FT, et al. Prevalence of osteoporosis and osteopenia in elderly patients scheduled for total knee arthroplasty. Arch Orthop Trauma Surg. 2022;142(12):3957-64.

8. Zhao AY, Agarwal AR, Harris AB, Cohen JS, Golladay GJ, Thakkar SC. The Association of Prior Fragility Fractures on 8-Year Periprosthetic Fracture Risk Following Total Hip Arthroplasty. J Arthroplasty. 2023;38(7 Suppl 2):S265-S9.e5.

9. Kubo M, Nosaka Y, Hasegawa T, Kumagai K, Amano Y, Isoya E, et al. Osteoporosis should be evaluated by bone mineral density at the combination of the lumbar spine and ipsilateral femoral neck in female patients with knee osteoarthritis scheduled for knee arthroplasty: A retrospective observational study. J Orthop Sci. 2024.

10. Shepard S, Bartholomew A, Houserman D, Bamberger HB, Manocchio AG. Assessing osteoporosis screening compliance in total joint surgery: a retrospective chart review. J Osteopath Med. 2024;124(12):537-41.

11. Murahashi Y, Teramoto A, Jimbo S, Okada Y, Kamiya T, Imamura R, et al. Denosumab prevents periprosthetic bone mineral density loss in the tibial metaphysis in total knee arthroplasty. Knee. 2020;27(2):580-6.

12. Wang CJ, Wang JW, Weng LH, Hsu CC, Huang CC, Chen HS. The effect of alendronate on bone mineral density in the distal part of the femur and proximal part of the tibia after total knee arthroplasty. J Bone Joint Surg Am. 2003;85(11):2121-6.

13. Wang CJ, Wang JW, Ko JY, Weng LH, Huang CC. Three-year changes in bone mineral density around the knee after a six-month course of oral alendronate following total knee arthroplasty. A prospective, randomized study. J Bone Joint Surg Am. 2006;88(2):267-72.
14. Yamasaki S, Masuhara K, Yamaguchi K, Nakai T, Fuji T, Seino Y. Risedronate reduces postoperative bone resorption after cementless total hip arthroplasty. Osteoporos Int. 2007;18(7):1009-15.
15. Iwamoto N, Inaba Y, Kobayashi N, Ishida T, Yukizawa Y, Saito T. A comparison of the effects of alendronate and alfacalcidol on bone mineral density around the femoral implant and in the lumbar spine after total hip arthroplasty. J Bone Joint Surg Am. 2011;93(13):1203-9.
16. Iwamoto N, Inaba Y, Kobayashi N, Yukizawa Y, Ike H, Ishida T, et al. The effectiveness of mono or combined osteoporosis drug therapy against bone mineral density loss around femoral implants after total hip arthroplasty. J Bone Miner Metab. 2014;32(5):539-44.
17. Aro E, Moritz N, Mattila K, Aro HT. A long-lasting bisphosphonate partially protects periprosthetic bone, but does not enhance initial stability of uncemented femoral stems: A randomized placebo-controlled trial of women undergoing total hip arthroplasty. J Biomech. 2018;75:35-45.
18. Aro HT, Nazari-Farsani S, Vuopio M, Löyttyniemi E, Mattila K. Effect of Denosumab on Femoral Periprosthetic BMD and Early Femoral Stem Subsidence in Postmenopausal Women Undergoing Cementless Total Hip Arthroplasty. JBMR Plus. 2019;3(10):e10217.
19. Nakura N, Hirakawa K, Takayanagi S, Mihara M. Denosumab prevented periprosthetic bone resorption better than risedronate after total hip arthroplasty. J Bone Miner Metab. 2023;41(2):239-47.
20. Khatod M, Inacio MC, Dell RM, Bini SA, Paxton EW, Namba RS. Association of Bisphosphonate Use and Risk of Revision After THA: Outcomes From a US Total Joint Replacement Registry. Clin Orthop Relat Res. 2015;473(11):3412-20.
21. Namba RS, Inacio MC, Cheetham TC, Dell RM, Paxton EW, Khatod MX. Lower Total Knee Arthroplasty Revision Risk Associated With Bisphosphonate Use, Even in Patients With Normal Bone Density. J Arthroplasty. 2016;31(2):537-41.
22. Forlenza EM, Serino J, 3rd, Acuña AJ, Terhune EB, Behery OA, Della Valle CJ. Bisphosphonate Use in Patients Who Have Osteoporosis Does Not Increase the Risk of Periprosthetic Fracture Following Total Knee Arthroplasty. J Arthroplasty. 2024.
23. Serino J, 3rd, Terhune EB, Harkin WE, Weintraub MT, Baim S, Della Valle CJ. Bisphosphonate Use May be Associated With an Increased Risk of Periprosthetic Hip Fracture. J Arthroplasty. 2024;39(2):448-51.e1.
